# Regional variations in human milk oligosaccharides in Vietnam suggest FucTx activity besides FucT2 and FucT3

**DOI:** 10.1038/s41598-018-34882-x

**Published:** 2018-11-14

**Authors:** Sander S. van Leeuwen, Eline Stoutjesdijk, Geert A. ten Kate, Anne Schaafsma, Janneke Dijck-Brouwer, Frits A. J. Muskiet, Lubbert Dijkhuizen

**Affiliations:** 1 Microbial Physiology, Groningen Biomolecular Sciences and Biotechnology Institute (GBB), University of Groningen, Nijenborgh 7, 9747 AG Groningen, The Netherlands; 2 Department of Laboratory Medicine, University Medical Center Groningen, University of Groningen, 9713 GZ Groningen, The Netherlands; 3 FrieslandCampina, Stationsplein 4, 3818 LE Amersfoort, The Netherlands; 4 Present Address: Department of Laboratory Medicine, University Medical Center Groningen, University of Groningen, 9713 GZ Groningen, The Netherlands; 5 Present Address: CarbExplore Research BV, Zernikepark 12, 9747 AN Groningen, The Netherlands

## Abstract

Breastfeeding is the normal way of providing young infants with the nutrients they need for healthy growth and development (WHO). Human milk oligosaccharides (*h*MOS) constitute a highly important class of nutrients that are attracting strong attention in recent years. Several studies have indicated that *h*MOS have prebiotic properties, but also are effective in anti-adhesion of pathogens, modulating the immune system and providing nutrients for brain growth and development. Most of the latter functions seem to be linked to the presence of fucose-containing immunodeterminant epitopes, and Neu5Ac-bearing oligosaccharides. Analysis of *h*MOS isolated from 101 mothers’ milk showed regional variation in Lewis- and Secretor based immunodeterminants. Lewis-negative milk groups could be sub-divided into two sub-groups, based on the activity of a third and hitherto unidentified fucosyltransferase enzyme. Analysis of *h*MOS remaining in faeces showed three sub-groups based on *h*MOS surviving passage through the gut, full consumption, specific partial consumption and non-specific partial consumption, fitting previous findings.

## Introduction

Human mature milk contains about 70 g/L lactose^1^, and 5–15 g/L oligosaccharides, of which ~10% are acidic oligosaccharides^2^. Human milk oligosaccharides (*h*MOS) have been indicated to have diverse effects on health and growth of the infant^3^. Observed activities include immune-stimulating effects^4^, influence on brain development^5^, local effects in the gastro-intestinal tract^6^, anti-adhesive properties^7–9^, and modifying composition of microbiota (prebiotics)^10–12^. Many of the beneficial effects, other than prebiotic properties, are often, but not exclusively, attributed to structures bearing fucose, sialic acid or both^2^.

**Figure 1:**
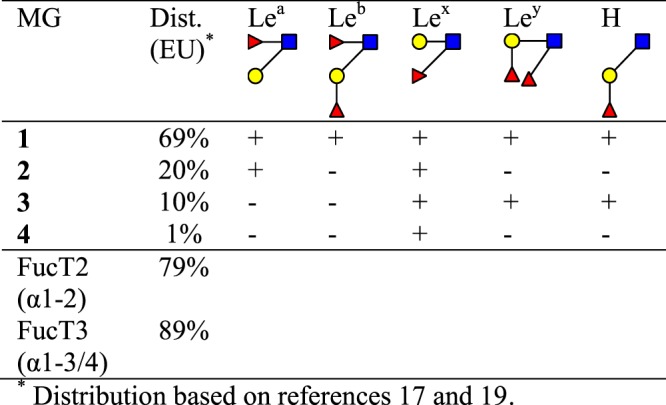
Expected structural epitopes and relative occurrence are indicated for the four milk groups (MG). The bottom part shows the derived presence of actively expressed FucT2 and FucT3 enzymes.

Over two hundred different *h*MOS have been reported and more than one hundred have been structurally characterized^2,13–17^. Important to note is that not every woman synthesizes the same ensemble of oligosaccharides. Particularly the fucosylation of *h*MOS differs per individual, mirroring the Secretor status and Lewis blood group of the mother. Activity of two fucosyltransferase encoding genes results in the occurrence of four milk groups. The genes in question are the Secretor (*Se*) gene *fut2*, coding for α-1,2-fucosyltransferase (FucT2), and the Lewis (*Le*) gene *fut3*, coding for α-1,3/1,4-fucosyltransferase (FucT3) (Fig. 1)^2,9,15,18–20^. For *fut2* a wide range of both functional and non-functional alleles have been described^21^. The occurrence of the various alleles are racially diverse^22,23^. For *fut3* the amount of non-functional alleles is more limited, but a broad range of functional alleles is known. Also, for *fut3* specific racial populations show occurrence of specific sub-sets of SNPs^24,25^. Furthermore, besides FucT3, also the *Se*- and *Le*-independent α-1,3-fucosyltransferases FucT4, 5, 6, 7, and/or 9 may play a role in the final ensemble of Fuc-containing *h*MOS^9^.

Recently we have published an NMR based method to determine milk groups, based on specific reporter-group signals in 1D ^1^H NMR spectra^20^. Here we describe the application of the previously developed method on milk samples and their corresponding infant faecal samples in different regions of Vietnam (~20 samples per region). The samples were collected in Ha Long Bay (HB), Tien Giang (TG), Phu Tho (PT), Ho Chi Minh (HC) and Ha Noi (HN) (Fig. S1). Vietnam is a diverse country in terms of geography (long North-South distance), genetic backgrounds, and cultural habits including diet. The five selected regions are based on a) geography; HB, HN and TG in the North, with TG inland and HB on the coast, while HC and PT are situated in the South, and b) general lifestyle; HN and HC are large cities, providing urbanized conditions and possibly resulting in different dietary habits, while HB, PT and TG are more rural areas.

These differences in living and dietary habits may have a significant influence on the composition of the milk. In this study we investigate the composition of *h*MOS under these different conditions. Secondly, we investigate how these *h*MOS of different compositions survive passage through the infant digestive tract. In terms of fucosylation of *h*MOS interesting differences have been observed between the regions. In the total sample set all 4 milk groups have been observed, however, the balance between the groups differs between the regions. Faeces samples showed three types of *h*MOS consumption, i) complete consumption, ii) specific consumption and iii) non-specific consumption.

## Results

### Milk group classification

**Figure 2:**
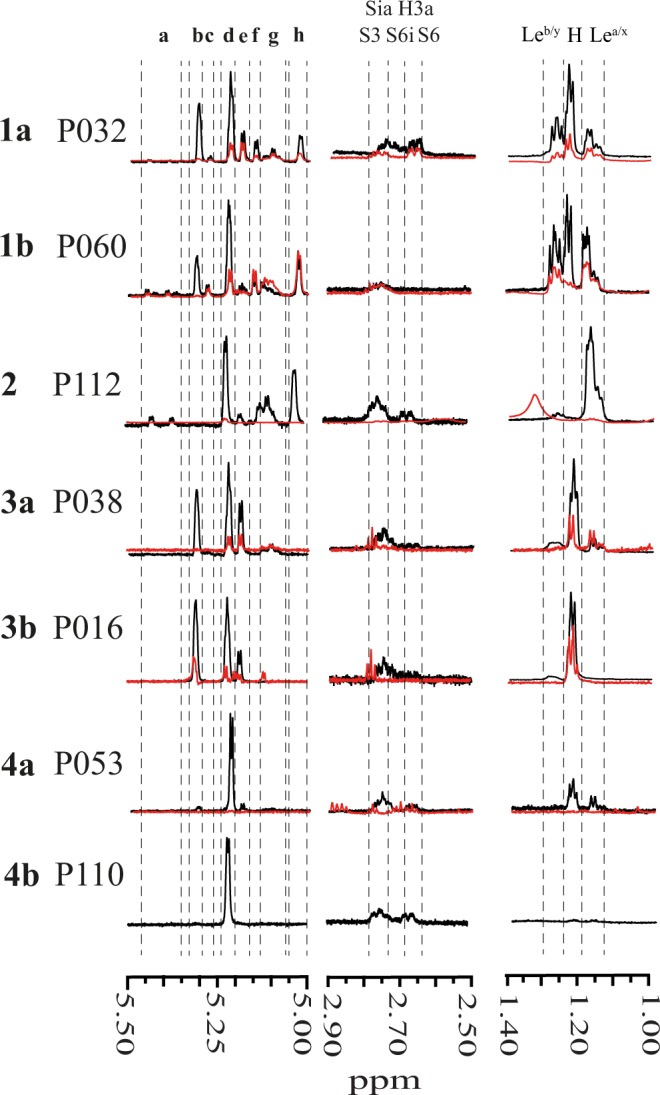
Example 1D ^1^H NMR spectra for milk groups **1a**, **1b**, **2**, **3a**, **3b**, **4a** and **4b**. Black line is the spectrum for mothers’ milk and the red line (where present) is for the corresponding infant faeces sample. Structural-reporter-group signals are indicated; anomeric region δ5.00–5.50: (**a**) Fuc(α1–3)- H-1 in pseudo-Le^x^ (3-FL) and pseudo-Le^y^ (DF-L) epitopes, (**b**) Fuc(α1–2)- H-1 in 2′-FL, (**c**) Fuc(α1–2)- H-1 in Le^y^ epitopes, (**d**) α-D-Glc*p* H-1, (**e**) Fuc(α1–2)- H-1 in H-antigen epitopes and α-D-Glc*p* H-1 in 3-FL and DF-L, (**f**) Fuc(α1–2)- H-1 in Le^b^ epitopes, (**g**) Fuc(α1–3)- H-1 in Le^x^ and Le^y^ epitopes (**h**). Fuc(α1–4)- in Le^a^ and Le^b^ epitopes, Neu5Ac region δ2.50–2.90 ppm: S3 Neu5Ac(α2–3)- H-3e, S6 Neu5Ac(α2–6)-Gal H-3e, and S6i Neu5Ac(α2–6)-GlcNAc H-3e; Fuc CH_3_ region δ 1.00–1.40: Le^b/y^ CH_3_ signals of Fuc residues in Le^b^ and Le^y^ epitopes, H, CH_3_ signals of Fuc residues in H-antigen epitopes and Le^a/x^, CH_3_ signals of Fuc residues in Le^a^ and Le^x^ epitopes.

Using the FucT2 and FucT3 dependent immuno-determinant milk group classification system (Fig. 1), samples were put into milk groups **1**, **2**, **3** or **4** based on the 1D ^1^H NMR spectrum (Figs 2 and S2; Table S1)^20^. Secretor status can be derived from the presence of Fuc(α1–2) H-1 signals (Figs 2 and S2) in the anomeric regions **b**, **c** and **e**. The presence of FucT3 activity (Lewis status) is derived from anomeric region h which is specific for Fuc(α1–4)- epitopes. There are two brackets containing Fuc(α1–3)-related signals; anomeric region **a** for 3-FL and LDFT structures, and anomeric region **g** for all other Le^x^ and Le^y^ derived Fuc(α1–3)- residues^20^. Individuals that have significant peaks in one or more of the regions **b**, **c**, and **e** are classed as Secretors, absence of peaks in all three regions is the hallmark of non-Secretor individuals. Lewis-negative individuals will show no signals in region **h**, but still have a peak in region **g**. A Lewis-negative non-Secretor individual would only have peaks in regions **a**, **d** and **g**, while a Lewis-negative Secretor will have peaks in regions **b** and **e** for H-antigens, but not in region **c** for Le^b/y^ derived Fuc(α1–2) signals. A Lewis-positive Secretor would have signals in all anomeric regions.

**Figure 3:**
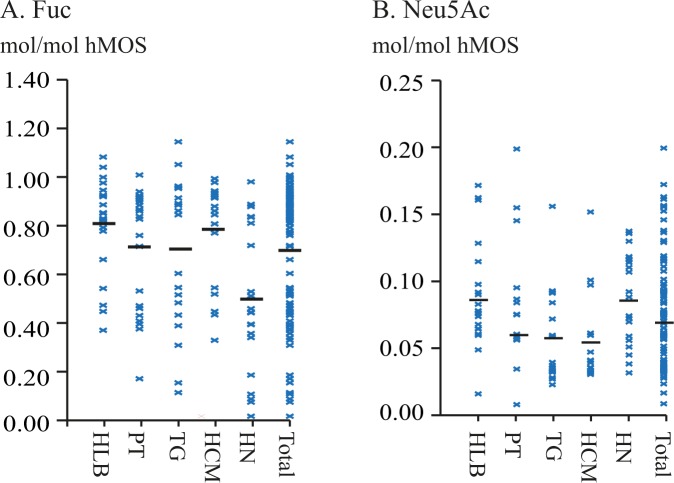
Molar-ratio distributions for the different regions and the total Vietnamese population of A. Fuc and B. Neu5Ac, in mol/mol *h*MOS based on HPAEC-PAD peak integrations of known peaks. Level of Fuc is based on peaks 1–7 and 10 in relation to all 14 peaks. Levels of Neu5Ac is based on peaks 10–14 in relation to all 14 peaks. Di-fucosylated structures count for 2 Fuc and structure 14 counts for 2 Neu5Ac.

These classifications were supported by HPAEC-PAD analysis (Figs 3 and S3), showing only structures containing the expected Fuc epitopes. Based on the Secretor/Lewis ratio (S/L) (Table S1) samples from milk group **1** were sub-divided into **1a** (S/L ≤ 1.50) and **1b** (S/L > 1.50)^20^. A total of 54 samples were categorised as milk group **1**, 40 into **1a** and 14 into **1b**. A total of 34 individuals showed a milk group **2** pattern. Milk groups **3** and **4** were represented by 7 and 6 individuals, respectively. In milk groups **3** and **4**, however, some interesting observations were made.

### Notable samples in milk groups 3 and 4

The classification into milk group **3** is based on the presence of a strong H-antigen related peak in the Fuc CH_3_ region, combined with the absence of a Lewis Fuc(α1–4)- anomeric signal (δ 5.00–5.06). Out of 7 samples 4 (M020, M038, M088, and M097) followed the pattern observed previously^20^, showing Fuc(α1–3)- residues in Le^x^ and Le^y^ epitopes, as evidenced by peaks in anomeric region **g** of the anomeric range, as well as peaks in the CH_3_ regions fitting with Le^a/x^ and Le^b/y^. Three samples (M016, M058, and M072) showed a different pattern, in which the **g** region, as well as the Le^a/x^ and Le^b/y^ CH_3_ regions were empty of peaks, showing only H-antigen CH_3_, together with 2′-FL (anomeric region **b**) and H-antigen (anomeric region **e**) related anomeric signals. In this study 6 samples were classifieed as milk group **4** (M030, M053, M071, M110, M111, and M116), i.e. Lewis-negative non-Secretor type. In the case of M110 no peaks belonging to Fuc were observed. The Fuc CH_3_ region showed no peaks, and the δ 5.00–5.50 ppm region only showed a Glc H-1 peak (anomeric region **d**). There were *h*MOS present in this sample, however, as can be deduced from the presence of *N*-acetyl CH_3_, as well as Neu5Ac H3a signals.

### Regional differences in FucT deficiencies

Using the 1D ^1^H NMR spectra, as described above, the 101 samples analysed were finally classified into milk groups **1a**, **1b** (54 samples, in total 53.4%; 39.6% **1a**, and 13.8% **1b**), milk group **2** (34 samples, 33.7%), milk group **3** (7 samples, 7.0%) and milk group **4** (6 samples, 5.9%) (Table 1).

**Table 1:** Milk group (MG) distribution and FucT (FT#) activity for all regions of Vietnam and the total sample population in %. Milk group 1 is separated into 1a and 1b based on Fuc(α1–2) and Fuc(α1–3/4) ratio as described (19), milk groups 3 and 4 are separated into subgroups based on the presence (a) and absence (b) of Fuc(α1–3)- epitopes.

MG	HB n = 20	PT n = 22	TG n = 20	HC n = 18	HN n = 21	Total n = 101
1a	30.0	50.0	35.0	50.0	33.3	39.6
1b	40.0	9.1	10.0	11.1	0.0	13.8
2	20.0	31.8	35.0	27.8	52.4	33.7
3a	5.0	4.5	0.0	11.1	0.0	4.0
3b	5.0	0.0	10.0	0.0	0.0	3.0
4a	0.0	4.5	10.0	0.0	9.5	4.9
4b	0.0	0.0	0.0	0.0	4.8	1.0
FT2	80.0	63.6	55.0	72.2	33.3	60.4
FT3	90.0	90.9	80.0	88.9	85.7	87.1
FTx	95.0	100.0	90.0	100.0	95.2	96.0

Notable differences were observed per region, however. In Ha Long Bay-region (HB; n = 20) the distribution of milk groups resembled the distribution found in Europe (Fig. 1) very closely. The Ha Noi-region (HN; n = 22) had the highest incidence of *fut2* deficiency, with 33% Secretor type individuals, while HB-region with 80% Secretor individuals had 20% *fut2* deficient individuals. For *fut3* the spread was from 80% Lewis-positive in Tien Giang-region (TG; n = 20) with lowest occurrence up to 90% in Ha Long Bay region.

### HPAEC-PAD analysis

**Figure 4:**
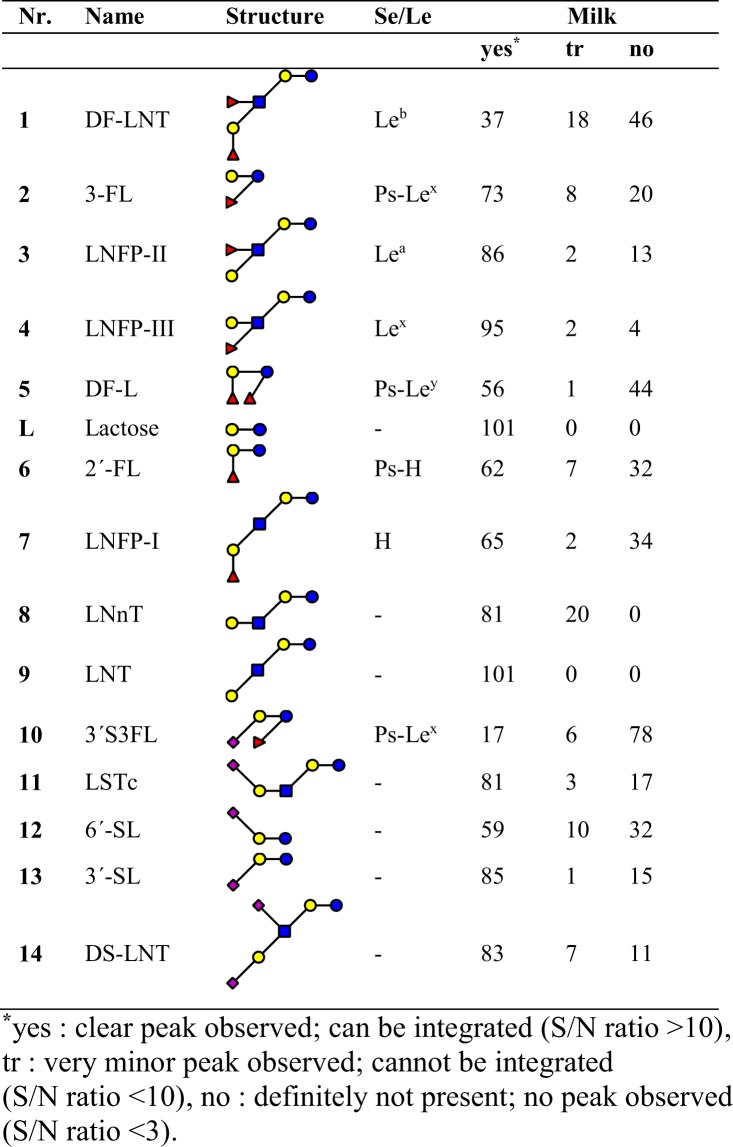
HPAEC-PAD analysis of 14 reference compounds. Secretor and Lewis based histo-blood groups. The occurrence in milk samples based HPAEC-PAD analysis is shown.

HPAEC-PAD profiling (Figs 4 and S3) was used to fingerprint the milk samples based on 14 known reference structures. All samples showed a residual lactose peak, but also lacto-N-tetraose (LNT) and lacto-N-neotetraose (LNnT) were observed in all samples (Fig. 4). Except for 3′-S3-FL all acidic structures followed were observed in most samples. Using HPAEC-PAD analysis (Figs 4 and S3) 14 reference structures could be followed, bearing specific FucT2 and FucT3 dependent structural elements. Analysis of these data supported the milk group classification made by NMR spectroscopy.

Based on the 14 followed structures the levels of Fuc and Neu5Ac could be estimated. Peaks of fucosylated structures were integrated and standardized to the fucose standard (200 μM) peak. Monofucosylated structures counted towards one fucose, difucosylated peaks counted for two fucose redisues. Comparison of the total fucose residues derived from these peaks and the total *h*MOS derived from the levels of all integrated peaks, excluding lactose, yielded roughly estimated levels of fucose-residues expressed in mol Fuc/mol *h*MOS (Fig. 3). The Fuc:*h*MOS molar ratio showed a broad distribution (Fig. 3A), with an average at 0.69 mol Fuc/mol *h*MOS.

**Figure 5:**
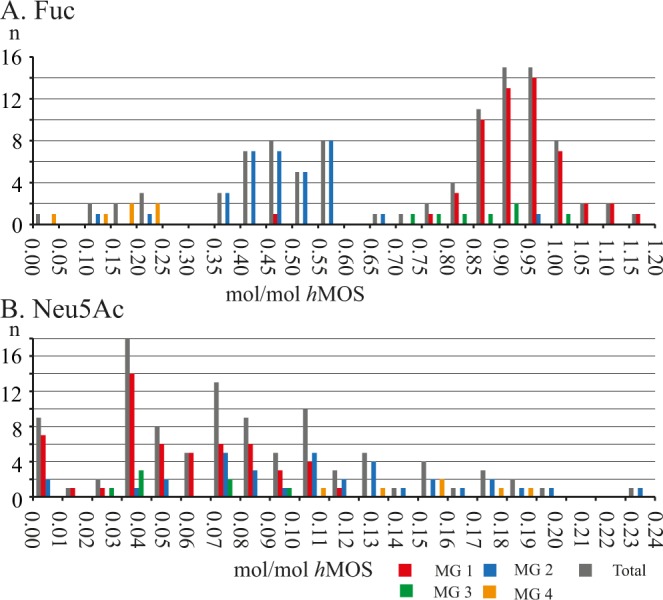
Distribution plots of (**A**) Fuc and (**B**) Neu5Ac in mol/mol *h*MOS derived from HPAEC-PAD for the total sample population (grey), compared with milk group **1** (red), milk group **2** (blue), milk group **3** (green) and milk group **4** (orange).

Plotting the Fuc distribution (Fig. 5A) for the total sample population (grey bars) yielded a double distribution, between 0.30 and 0.60 mol Fuc/mol *h*MOS, with milk group **2** (blue bars) and between 0.75 to 1.20 mol Fuc/mol *h*MOS, with milk groups **1** (red bars) and **3** (green bars). Milk group **4** samples (n = 6) all displayed very low levels of Fuc between 0.00 and 0.25 mol Fuc/mol *h*MOS, together with two outliers from milk group **2**.

Considering the Vietnam regions sampled, the lowest average Fuc levels were observed in Ha Noi (0.49 mol Fuc/mol *h*MOS) and the highest average levels in Ha Long Bay and Ho Chi Mihn (0.80 and 0.78 mol Fuc/mol *h*MOS, respectively). The lower abundance of Fuc in Ha Noi coincided with the higher representation of women in milk group **2**. In all regions the spread in distribution of Fuc levels was broad (Fig. 3A), ranging between 0.38 and 1.11 mol/mol *h*MOS in Ha Long Bay and between 0.00 and 1.01 mol/mol *h*MOS in Ha Noi.

NMR spectroscopic analysis only provided an approximate indication of Neu5Ac levels, ranging from trace amounts (e.g. M056, M059 and M085) up to significant peaks in (e.g. M011 and M039). NMR analysis did allow determination of the ratio between Neu5Ac(α2–3)- (**S3**) and Neu5Ac(α2–6)- (**S6**) epitopes. Residues of Neu5Ac that are (α2–3)- linked to Gal provide a broad peak in **S3** labeled region of the 1D ^1^H NMR spectrum (Figs 2 and S2). Similarly Neu5Ac that is (α2–6)-linked to Gal provides a peak in **S6**. In the area in between a peak can be observed for Neu5Ac that is (α2–6)-linked to GlcNAc residues in region **S6i**. The relative levels of two sub-categories of Neu5Ac(α2–6)-,i.e. between Neu5Ac(α2–6)Gal (**S6**) and Neu5Ac(α2–6)GlcNAc (**S6i**), can be estimated as well. Of the 101 samples studied, 57 (47.1%) showed higher or similar levels of (α2–3) compared to (α2–6)-linked Neu5Ac epitopes (Fig. S2). It is not entirely straightforward to determine this, as some internal Neu5Ac(α2–6)- (**S6i**) residues have an H3a signal overlapping with the Neu5Ac(α2–3)- (**S3**) H3a area. Furthermore, HPAEC-PAD analysis (Fig. S3) of samples containing clear peaks for sialyl-lactose revealed either a higher 3′-SL peak than a 6′-SL peak (in 21 samples) or that the 3′-SL and 6′-SL peaks were of similar intensity (26 samples). In 14 samples no sialyllactose was detected at all, and in another 7 samples no 6′-SL was detected, but 3′-SL was present in trace amounts. In all other samples 6′-SL was the predominant structure.

Using HPAEC-PAD peaks for mono- and di-sialylated structures in relation to all integrated *h*MOS to determine the relative levels of sialic acid as mol Neu5Ac/mol *h*MOS, the levels of Neu5Ac ranged from trace amounts up to 0.20 mol Neu5Ac/mol *h*MOS (Fig. 3). The regional average levels of Neu5Ac varied between 0.05 and 0.10 mol Neu5Ac/mol *h*MOS (Fig. 3B). The spread of data, however, was broad in all regions. Plotting a distribution (Fig. 5B) for sialic acid levels showed a non-Gaussian distribution when observing the total test population (grey bars). Splitting the data by milk group showed separate distributions for milk group **1** (red bars), between 0.00 and 0.10 mol Neu5Ac/mol *h*MOS, and milk group **2** (blue bars), between 0.02 and 0.17 mol Neu5Ac/mol *h*MOS. The number of samples in milk groups **3** (green bars) and **4** (purple bars) were too low to show a clear distribution. It is evident, however, that the milk group **3** samples are all in the lower half of the distribution, while milk group **4** samples are all in the higher half. Plotting distributions by region (not shown), rather than milk group, does not show a Gaussian distribution.

### Faecal analysis

Analysis of NMR spectra of faecal samples showed three types of profiles (Figs 2 and S2). In the first profile all structural elements found in the milk samples were still present in the corresponding faeces samples (Fig. 2; spectra F016 and F032) (19 out of 76 samples). Often minor changes in relative intensities occurred, indicating the faecal *h*MOS composition is different from the milk. In the second profile all *h*MOS structures were absent in the faecal samples (Fig. 2; spectra F053 and F112) (20 out of 76 samples). In the third profile specific structural epitopes in the milk *h*MOS were no longer present, or greatly reduced in the faeces (Fig. 2; spectra F038 and F060) (37 out of 76 samples). Milk group distribution of the milk in the milk-faeces sample sets was 43 (56.6%) milk group **1**, 24 (31.6%) milk group **2**, 6 (7.9%) milk group **3** and 3 (3.9%) milk group **4**. Notably, in 30 out of 37 cases of specific structural epitope consumption occurred in milk group **1** related samples. In case of milk group **2** related samples 13 out of 24 cases showed all structural elements in the faeces that were also observed in the milk.

## Discussion

### Exclusivity of breastfeeding

From all participants 69.3% provided exclusive breastfeeding during the sampling period (Table S2). Several participants did not fill out the food questionnaire (7.9%). The other 22.8% provided mixed feeding of which breastmilk was a significant part of the diet. In most cases (17.8%) the feeding was mixed with formula. In 2.0% of the cases other liquids, e.g. water or juice, was added to the diet, in 2.0% of the cases mashed solids were already added to the diet and in 1 case formula as well as other liquids were added (1.0%). In the composition of the *h*MOS in mothers’ milk and in the *h*MOS patterns found in the faecal samples no correlations were observed specifically with the different mixed feedings.

### Milk group distribution

Milk groups were assigned based on 1D ^1^H NMR profiles (Table 1 and Fig. S2) and verified by HPAEC-PAD profiling (Figs 4 and S3). The presence of 2′-FL was observed in 69 samples, 62 times with a significant peak, 7 times in trace amounts (M053, M056, M061, M071, M102, M104 and M105); i.e. peak height <10x noise. Another Se-specific structure, LNFP I was observed in 67 milk samples, 65 times with a clear peak and 2 times (M001 and M071) in trace amounts. This presence is more frequent than the amount of Secretor individuals. Closer inspection of the HPAEC-PAD profiles (Fig. S3) shows that samples sorted as non-Secretor with detected presence of 2′-FL and LNFP I all had very minor peaks of these structures. These are probably the result of small levels of FucT1 activity^2,3,9^.

Classification of milk samples into four Lewis-Secretor histo-blood group based milk groups resulted in identification of four Lewis-negative individuals (M016, M058, M072 and M110) in milk groups **3** and **4** that showed a pattern not observed previously, i.e. no Fuc(α1–3)- epitopes^9,19^. A study on *h*MOS levels in different milk groups showed that the level of Le^x^-containing *h*MOS are similar between Lewis-positive and Lewis-negative individuals^19^. These results suggest that the back-up FucT enzymes for Le^x^ and Le^y^ epitopes in these individuals also were disabled^9^. It is very unlikely that all enzymes FucT4, 5, 6, 7 and 9 are disabled via a SNP or unequal cross-over mutations. Therefore we propose that only one enzyme, FucTx is responsible for the synthesis of Fuc(α1–3)- containing structures in FucT3 deficient individuals. This observation divides milk groups **3** and **4** into two sub-groups based on the activity of FucTx, whereby the subgroup showing evidence of FucTx activity is classed as **3a**, and **4a**, respectively and the subgroup showing deficiency in FucTx is classed as **3b** and **4b**, respectively. One of the early papers on *h*MOS showed that a few individuals that were FucT3 deficient were also incapable of producing LNFP III^26^. While LNFP III is one of the most abundant Le^x^ structures, no evidence for 3-FL or LDFT was observed either, it was not possible to conclude a complete absence of the Le^x^ epitope at this time. It seems likely that the individuals described in that study were also FucTx deficient. The individuals sampled in this study showing lack of FucTx activity may be interesting in identifying the FucT-enzyme is responsible for this activity.

### Regional variations

Observations on specific structural elements from 1D ^1^H NMR (Fig. S2), as well as specific reference structures 1–14 (Figs 4 and S3), showed no remarkable differences in *h*MOS composition between regions. There were no specific structures that showed an up- or down-regulation in any of the regions. Although we initially hypothesized that cultural and dietary habits might influence the *h*MOS composition of milk, all variations we observed could be explained by the genetics of FucT2 and FucT3 coding genes.

In this study a very strong variation in the occurrence of the four milk groups was observed between the regions sampled. The milk group distribution translates into a level of FucT2 and FucT3 activity within the different sub-populations (Table 1). There is a certain racial diversity in which SNPs occur in the *fut2* and *fut3* genes within a certain population^21–25^. Moreover, the relative level of incidence of these SNPs and thereby the level of non-Secretor or Lewis-negative individuals also varies, due to differential levels of non-functional allele occurrence^27^. In the *fut2* gene, a non-functional unequal cross-over was observed in Japan^28^, and later also in Japan two different types of non-functional fusion genes were found^29^. Among different sub-populations within China, different occurrences of SNPs in *fut2* have been observed^22^. Although the levels of non-Secretor and Lewis-negative incidences have not been derived from these genetic population analyses. A milk oligosaccharide based study in China including 520 individuals showed all to be able to synthesize 2′-FL, however in some cases at very low levels suggesting a secondary route in *fut2* deficient Han Chinese^30^. A comparative genetic analysis has shown *fut2* deficiency in Xhosa populations up to 16% and *fut3* deficiency up to 22%^23^. In that same study Caucasian individuals showed *fut2* and *fut3* deficiency prediction of 30% and 9%, respectively. The *fut2* deficiency levels is in contrast with other phenotype derived studies^18–20^, while the *fut3* number for Caucasian populations fit with the phenotypical determinations. A recent study on the geographic differences in *h*MOS composition between Europe, North- and South America and several African countries showed levels of Secretor (*fut2* positive) individuals between 65% (Ethiopia and Gambia) up to 98% (Peru)^31^.

It is very well possible that the strong regional differences in milk groups observed in this study reflect the divergent ethnic backgrounds of people in Vietnam, with 54 ethnic groups present according to the Vietnamese census office.

### Fuc and Neu5Ac bearing hMOS

The estimated levels of Fuc and Neu5Ac in the milk samples showed a very broad distribution. In case of Fuc the levels in mol/mol *h*MOS showed strong correlation with the milk groups (Fig. 5A). Individuals that are FucT2 deficient (milk groups **2** and **4**) showed a lower average Fuc content in the milk. This fits with the observations in HPAEC-PAD that in milk group **1** individuals the 2′-FL and LNFP I peaks are often the highest peaks. This finding also fits with recent observations on Se/Le status on *h*MOS composition^32^. Also in studies on pooled milk the levels of 2′-FL and LNFP I are usually considered higher than LNT of LnNT^19,33^. When considering the different regions the lowest levels were observed in Ha Noi, which is also the region with the highest levels of milk group **2**, which may explain the relatively low average of Fuc. The two regions with the highest levels of Fuc, Ha Long Bay and Ho Chi Mihn (0.80 and 0.78 mol/mol *h*MOS, respectively) have also the highest occurrence of active FucT2 (80% and 72.2%, respectively). It seems that the levels of functional *fuc2* gene is the steering factor behind the Fuc levels in the human milk.

One structure of specific interest is LNFP III, containing the Le^x^ antigen, which is indicated in prevention of HIV adhesion to DC-SIGN^34^. A concentration-dependent protection against HIV transmission from mother to child has been observed in an African study^35^. In this study 95 individuals out of 101 have significant levels of this LNFP III, 2 more have trace amounts, and only 4 individuals, that are FucTx deficient, show a full absence of this structure.

Considering the levels of sialylation of *h*MOS there is a certain correlation between the Neu5Ac levels and Fuc-based milk groups. Although the average level of sialylation in milk group **2** is slightly higher than for milk group **1**, the distribution within milk group **2** is still very broad. Milk group **3** seems to be on the same level as milk group **1**, and milk group **4** shows Neu5Ac levels in the higher half of the spectrum. It should be noted that the quantity of samples in milk groups **3** and **4** is too limited for strong statements. These findings are in accordance with previous observations on effect of Secretor status on *h*MOS sialylation levels^19,32,36,37^. In principle, it would not be surprising to see an indirect effect of milk groups. Since some of the Neu5Ac containing *h*MOS are in competition for precursors with the FucT2 and FucT3 enzymes. A strong presence of Fuc, particularly in the form of LNFP I, LNFP II and LNFP III, could prevent effective sialylation into DSLNT or LST c, which are some of the more abundant acidic *h*MOS.

Interestingly, not all samples contain some of these more abundant sialylated *h*MOS. Of particular interest is the disialo-lacto-N-tetraose (DSLNT) structure, which has been indicated in a rat model study as a potentially important *h*MOS in prevention of necrotizing enterocolitis^38^. It should be noted that this has not been verified in a human based study. Necrotizing enterocolitis is a severe gut infection that occurs with relatively high frequency in preterm infants, with serious consequences. In this study 11 out of 101 mothers do not produce detectable levels of DSLNT and another 7 produce only trace amounts.

In terms of linkage types, Neu5Ac(α2–6)- epitopes are described to occur in higher abundance than Neu5Ac(α2–3)-^16,19,36,37^. In this study, however a significant number of individuals were observed where the Neu5Ac(α2–3)- levels were higher, or of similar intensity than Neu5Ac(α2–6)- levels. These observations showed that the statement about higher levels of (α2–6)-linked Neu5Ac in human milk^16^, is not a general rule. Perhaps individual variations in the levels of (α2–3) and (α2–6)-linked Neu5Ac have been missed in some previous studies^16,19,36,37^. It is also possible that in the Vietnamese population a different genetic control of sialylation occurs, resulting in a significant amount of individuals with a different (α2–3) to (α2–6) ratio in their *h*MOS. However, there are more individuals with a predominance of (α2–6)-linked sialic acid, which explains why pooled-milk studies would suggest such predominance as a general rule.

### Faecal analysis

The study of consumption of *h*MOS, determined by analysis of remaining *h*MOS in the faeces showed three types of patterns. There were individuals with a complete consumption of the *h*MOS in the milk, individuals where all structural elements in the milk were also observed in the faeces, be it with different relative intensities. Finally, individuals were observed in which one or more specific elements were completely or almost completely consumed, while others remained in the faeces. Previous studies on faecal *h*MOS have observed that there are differences between infants in *h*MOS composition leaving the gut over time^39–42^. One study on two infants showed that one baby had several *h*MOS still present in the faeces at 13 weeks of age, while the other had all *h*MOS still detected at 14 weeks^39^. Coupling the data to microbial taxa showed that the infant that retained all *h*MOS in the faeces had no detectable levels of Bifidobacteria in de faeces. Another study on 14 infants analysed at 2 and 7 months showed that at 2 months all infants still had *h*MOS in the faeces, while at 7 months 11 infants had no longer any detectable levels of *h*MOS and the other 3 had minor amounts of a few structures detectable^40^. The limitation of this study, however, is that only faeces were analysed, without comparing with the milk. Another draw-back of this study is the approach analysing only with MALDI-TOF-MS, detecting only compositional data, and no specific structures. A study with more time-points for analysis showed that in the first month all infants still had *h*MOS in the faeces^41^. As time progressed the composition of *h*MOS in faeces progressed through several stages towards further consumption. The amount of infants in this study was also relatively limited. Possibly the different types of faecal *h*MOS composition observed in our study was not observed in these studies due to limited cohort sizes. It is also possible that the occurrence at 1 month *post partum* of full-consumption type is specific to the Vietnamese cohort.

In almost all samples the relative amount of 2′-FL in the faeces is greatly reduced, except for one sample (F054) where there is a slight relative increase (Table S1). In total out of 76 samples 24 samples belong to milk group **2**, where 2′-FL is not present in the milk. In 49 cases the original milk did contain 2′-FL, but all 2′-FL was completely consumed in 40 cases. For the H-antigen only 18 cases are observed where this epitope is completely absent in the faeces when the corresponding milk sample contained a certain level of H-antigen. Previous studies have shown that several infant-gut related bacterial species can consume 2′-FL. It is therefore not surprising to see the relatively high levels of 2′-FL and H-antigen consumption^42–45^. It should be noted that a recent study showed a negative correlation between 2′-FL and levels of Bifidobacteria^42^. In that study 2′-FL correlated positively with Bacteroides levels instead.

When observing the consumption patterns in light of the milk groups, 30 out of 37 individuals with a consumption of a specific structural element, belonged to milk group **1**. It seems that many of the cases have a specific consumption of 2′-FL or H-antigen, which is not possible in milk group **2** individuals. The occurrence of complete consumption pattern showed no correlations with milk groups. It should be noted that too few samples in milk groups **3** and **4** prevent any strong statements to be made. There does not seem to be any specific correlation between consumption patterns and the different regions in Vietnam, when correcting for the differences in milk groups between regions.

## Conclusions

Using the 1D ^1^H NMR analytical approach developed previously^20^, the 101 samples could be classed into the milk groups. The Neu5Ac related peaks were of sufficient intensity to roughly elucidate the balance between Neu5Ac-(α2–3) vs Neu5Ac-(α2–6)- epitopes. Combining the NMR spectroscopy with HPAEC-PAD profiling allowed analysis of Neu5Ac and Fuc levels. Data showed an indication of minor differences in Neu5Ac and Fuc levels between the regions, but the spread of data points was large. Further studies with larger population sizes and better controlled diets would be interesting to further study this. More strong correlation was found between Neu5Ac and Fuc levels based on milk group classification of the samples. Milk groups **1** and **3** showed higher levels of Fuc, suggesting FucT2 to be more defining in Fuc levels than FucT3, while the relative levels of Neu5Ac was higher in case of milk groups **2** and **4**.

The distribution of milk groups observed in this study on samples from Vietnam is different than that observed in western studies. Mostly the level of non-Secretor individuals is higher in Vietnam than in western societies.

Finally, in contrast with all previous studies except for Kobata *et al*. (1969)^26^, here we find 4 individuals in Lewis-negative milk groups **3** and **4** that also show no Le^x^ and Le^y^ epitopes. These data lead to our hypothesis that only one enzyme, i.e. FucTx, is responsible for producing Le^x^ and Le^y^ epitopes in Lewis-negative (FucT3 inactive) individuals. Considering the fact that Secretor and Lewis status of an individual is the result of nonsense or missense SNPs in *fut2* and *fut3*, it seems likely that the observed FucTx deficiency is also the result of a SNP or unequal cross-over mutation, leading to a non-functional allele. With further study of the genetic make-up of this type of individuals, it may be possible to discover the enzyme responsible for the FucTx activity.

## Matrials and Methods

### Participants and sample collection

This cross-sectional study is part of the ‘ZOOG’ (‘Zonder Ontsteking Oud en Gezond’; *‘Without Infections Old and Healthy’*) project. The aim is to study relationships between maternal nutrient status, milk composition and maternal and offspring microbiome in various geographical regions with different cultural backgrounds. Apparently healthy and well-nourished lactating mothers, having at least one prior child, living in 5 provinces of Vietnam were invited to participate in the study. ‘Health and well nourishment’ were self-proclaimed and by visual observation. The study in Vietnam was approved by the Ethics Committee of The Family Food and Nutrition Institute in Hanoi, Vietnam. All women gave informed and written consent for participation of themselves, and for collection of data and faeces samples of their babies. The study was in agreement with the Helsinki declaration of 1975 as revised in 2013. Milk, blood and faecal samples were collected from the mother, faecal samples from the infant and blood and faecal samples from an elder sibling. The milk sample and faeces sample were taken at the same time, between 3 and 6 weeks post-partum. For this paper the milk samples (coded M###) and their corresponding infant faecal samples (coded F###) were used. Dietary and health information was collected via anthropometry questionnaire, health status questionnaire, social economic status questionnaire and 24 h recall for food consumption of lactating women were prepared by the National Institute of Nutrition, Hanoi, Vietnam.

### Sample handling

The mothers were instructed to save a 25 mL milk sample that was taken from a completely emptied breast around noon (10.00–14.00). All samples were collected in 10 mL aliquots and immediately frozen and stored at −80 °C (Vietnam). All samples were transported to the University Medical Center Groningen (the Netherlands) on dry ice. They were stored at −20 °C until transport to the lab. After transfer to the lab, samples were stored at −80 °C until further analysis. Prior to analysis samples were thawed at 4 °C overnight. A sample of 750 μL milk was diluted with 750 μL Milli-Q water, followed by centrifugation at 15000 × *g*, during 30 min. From the clear liquid phase, 1000 μL was isolated and transferred to a Carbograph SPE column (300 mg; Alltech) for *h*MOS extraction.

For 76 infants faeces corresponding with the milk were sampled in the morning prior to the milk sampling. For each sample ~500 mg of dry faecal matter was weighed and suspended in 2 mL, 20% vol/vol glycerol. The faecal suspension was centrifuged at 15000 × *g* for 30 min. Clear supernatant liquid was transferred to Carbograph SPE for *h*MOS extraction.

### Extraction of hMOS

Carbograph SPE columns (300 mg, Grace, Breda) were activated using 4.5 mL 80% acetonitrile, containing 0.1% TFA, washed with Milli-Q water, containing 0.1% TFA. Clear liquid from milk samples were applied and fully passed through the SPE column, followed by washing with 3 × 1.5 mL Milli-Q, and 3 × 1.5 mL 4% acetonitrile. Finally hMOS were eluted by 3 × 1.5 mL 40% acetonitrile containing 0.05% TFA. For faecal samples the columns were only washed with 3 × 1.5 mL Milli-Q water, followed by elution with 3 × 1.5 mL 40% acetonitrile, containing 0.05% TFA. After elution acetonitrile and TFA were evaporated by N_2_ stream and dried by lyophilization.

All samples were dissolved in exactly 1.00 +/− 0.05 mL Milli-Q water and a 100 μL aliquot was taken for HPAEC-PAD analysis. The remaining sample was used for NMR spectroscopy analysis.

### HPAEC-PAD analysis

Aliquots were lyophilized and re-dissolved in 1.00 +/− 0.05 mL Milli-Q containing 200 μM Fuc as internal standard for HPAEC-PAD profiling. HPAEC-PAD profiling was performed on a CarboPac PA-1 column (4 × 250 mm) at 1 mL/min, using a multi-step gradient of buffer A: 100 mM NaOH and B: 600 mM NaOAc in 100 mM NaOH; first 5 min 0% B, followed by three gradients of 15 min to 5% B, 15 min to 33.3% B, and 10 min to 66.6% B. Subsequently the column was washed for 7 min with 100% B followed by 8 min 0% B to equilibrate the column to starting conditions. A standard mixture of 14 commercially available *h*MOS (Fig. 4) was used to identify peak positions for the major *h*MOS. The reference mixture sample was analyzed once every 20 sample injections to verify stability in retention times between runs. All samples were analysed in duplicate on separate days.

### NMR spectroscopy

Samples were lyophilized and subsequently exchanged twice with 99.9%_atom_ D_2_O (Cambridge Isotope Ltd.). Finally samples were dissolved in 650 μL D_2_O containing internal acetone (δ^1^H 2.225). 600-MHz 1D ^1^H NMR spectra were recorded at 25 °C on a Varian Inova spectrometer (NMR department, University of Groningen) with 4800 Hz spectral width, collecting 16k complex points. The HOD signal was suppressed using a WET1D composite pulse with 10 Hz pulse-width. For each sample 16 cumulative transients were recorded. Processing of spectra was done using MestReNova 5.3 (Mestrelabs, Santiago de Compostella, Spain), applying automated phase correction and 5^th^ order polynomial baseline corrections, followed by manual spectral quality control. Integrations were done using pre-determined spectral binning regions based on previous studies^20^.

## Electronic supplementary material

Supplementary Information
