# Supplementary material for: Regional variations in human milk oligosaccharides in Vietnam suggest FucTx activity besides FucT2 and FucT3
Source: Sci Rep. 2018 Nov 14;8:16790. doi: 10.1038/s41598-018-34882-x (PMC6235895; doi:10.1038/s41598-018-34882-x)
Supplement: Supplementary file 1 — Supplementary Information [file 41598_2018_34882_MOESM1_ESM.pdf]

Supplementary Information belonging to:

Regional variations in human milk oligosaccharides in Vietnam suggest FucTx activity besides FucT2 and FucT3

Sander S. van Leeuwen,[1,*,a] Eline Stoutjesdijk,[2] Geert A. ten Kate,[1] Anne Schaafsma,[3] Janneke Dijck-Brouwer,[2] Frits A. J. Muskiet,[2] and Lubbert Dijkhuizen.[1,b]

[1] Microbial Physiology, Groningen Biomolecular Sciences and Biotechnology Institute (GBB), University of Groningen, Nijenborgh 7, 9747 AG, Groningen, The Netherlands

[2] Department of Laboratory Medicine, University Medical Center Groningen, University of Groningen, 9713 GZ Groningen, The Netherlands

[3] FrieslandCampina, Stationsplein 4, 3818 LE, Amersfoort, The Netherlands

[a]Current address: Department of Laboratory Medicine, University Medical Center Groningen, University of Groningen, 9713 GZ Groningen, The Netherlands

[b] Current address: CarbExplore Research BV, Zernikepark 12, 9747 AN Groningen, The Netherlands

[*] Corresponding author : e-mail, s.s.van.leeuwen@umcg.nl; Fax, +31 503611746

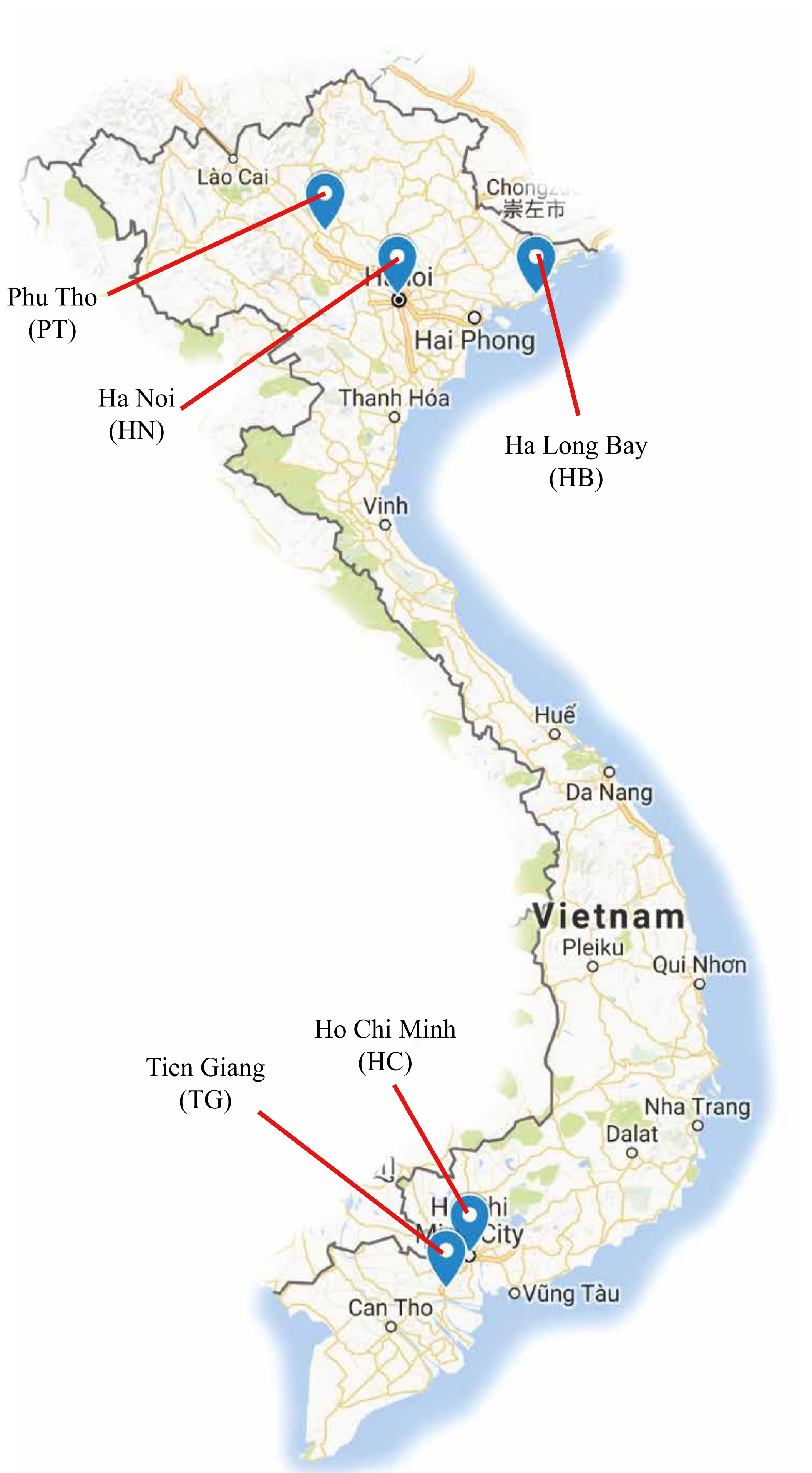

Map data © Google, SK telecom, ZENRIN

Fig. S1. Map of Vietnam indicating the 5 regions where participants were recruited. The map was derived from Google-maps (Google, SK telecom, ZENRIN) and modified in Adobe Photoschop CC2017.

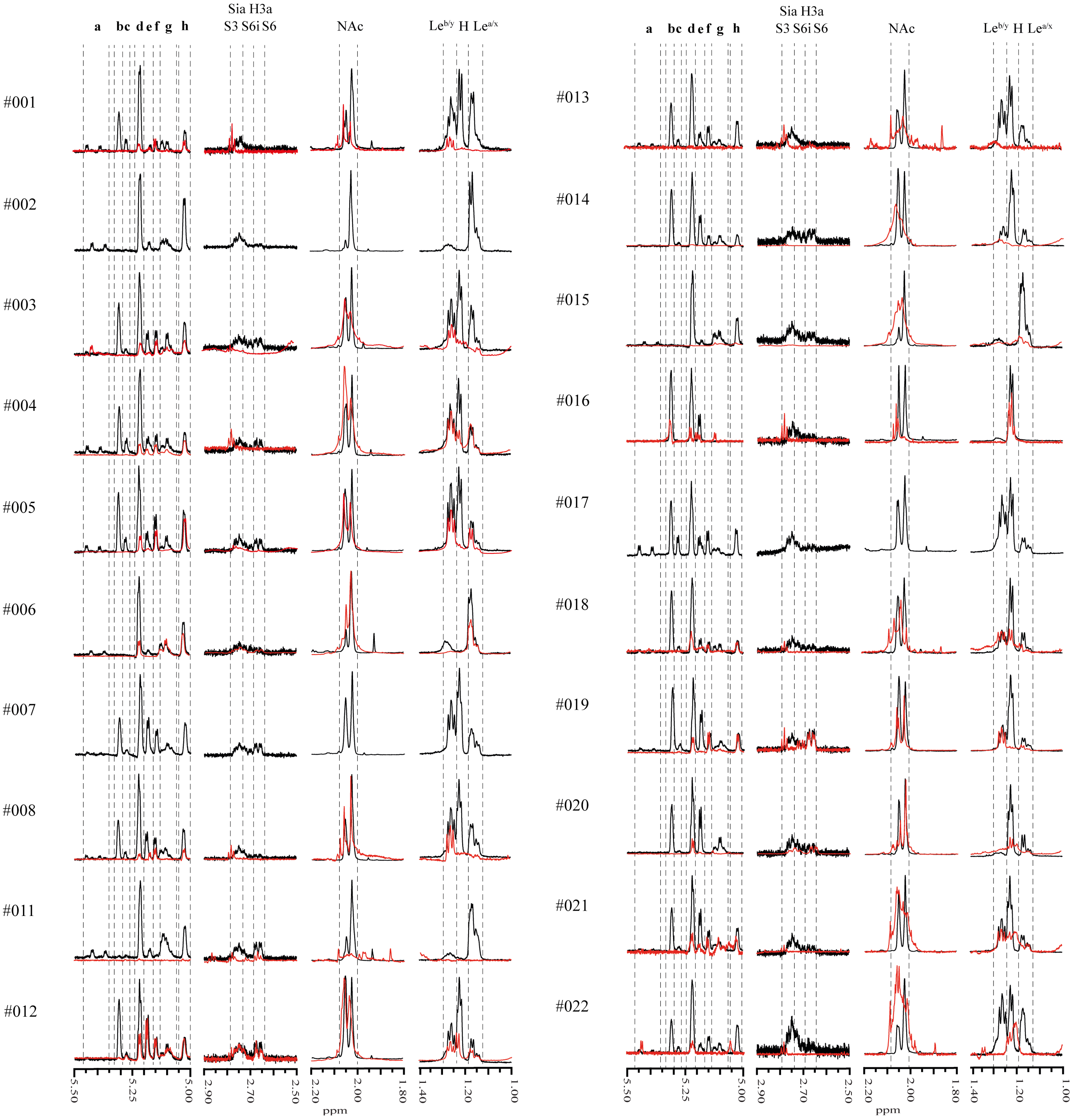

Fig. S2. Relevant parts of the 1D ^{1}H NMR spectra of human milk oligosaccharides isolated from mothers' milk (black) and corresponding faeces (red). Parts 1 – 5 show samples from 1. Ha Long Bay, 2. Phu Tho, 3. Tien Giang, 4. Ho Chi Mihn, 5. Ha Noi (Fig. S1).

(Fig. S2 – part 2)

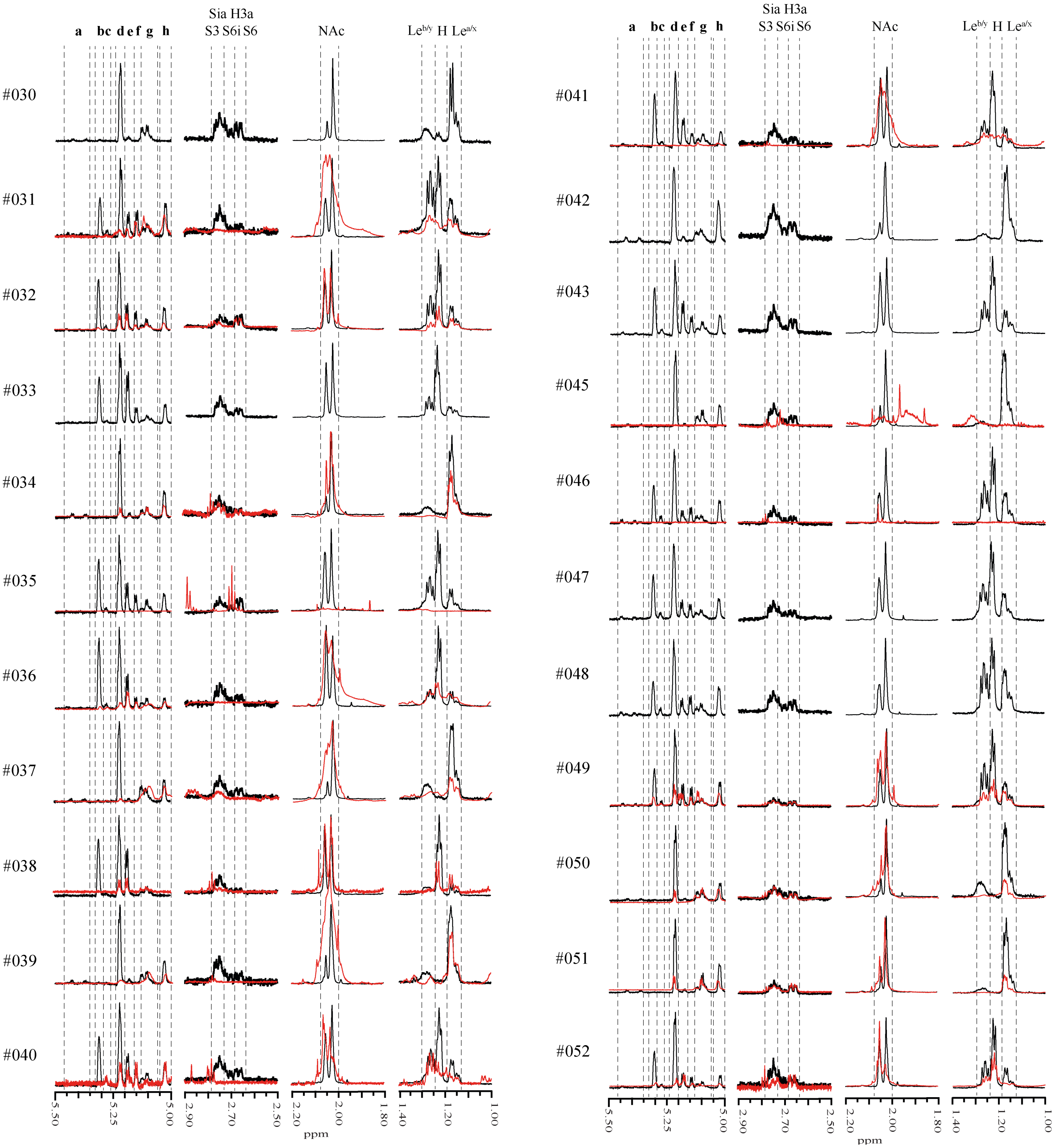
a
bc
d e f
g
h
Sia H3a
S3 S6i S6
NAc
Le$^{b/y}$ H Le$^{a/x}$
#030
#031
#032
#033
#034
#035
#036
#037
#038
#039
#040
#041
#042
#043
#045
#046
#047
#048
#049
#050
#051
#052
5.50
5.25
5.00
2.90
2.70
2.50
2.20
2.00
1.80
1.40
1.20
1.00
ppm

(Fig. S2 – part 3)

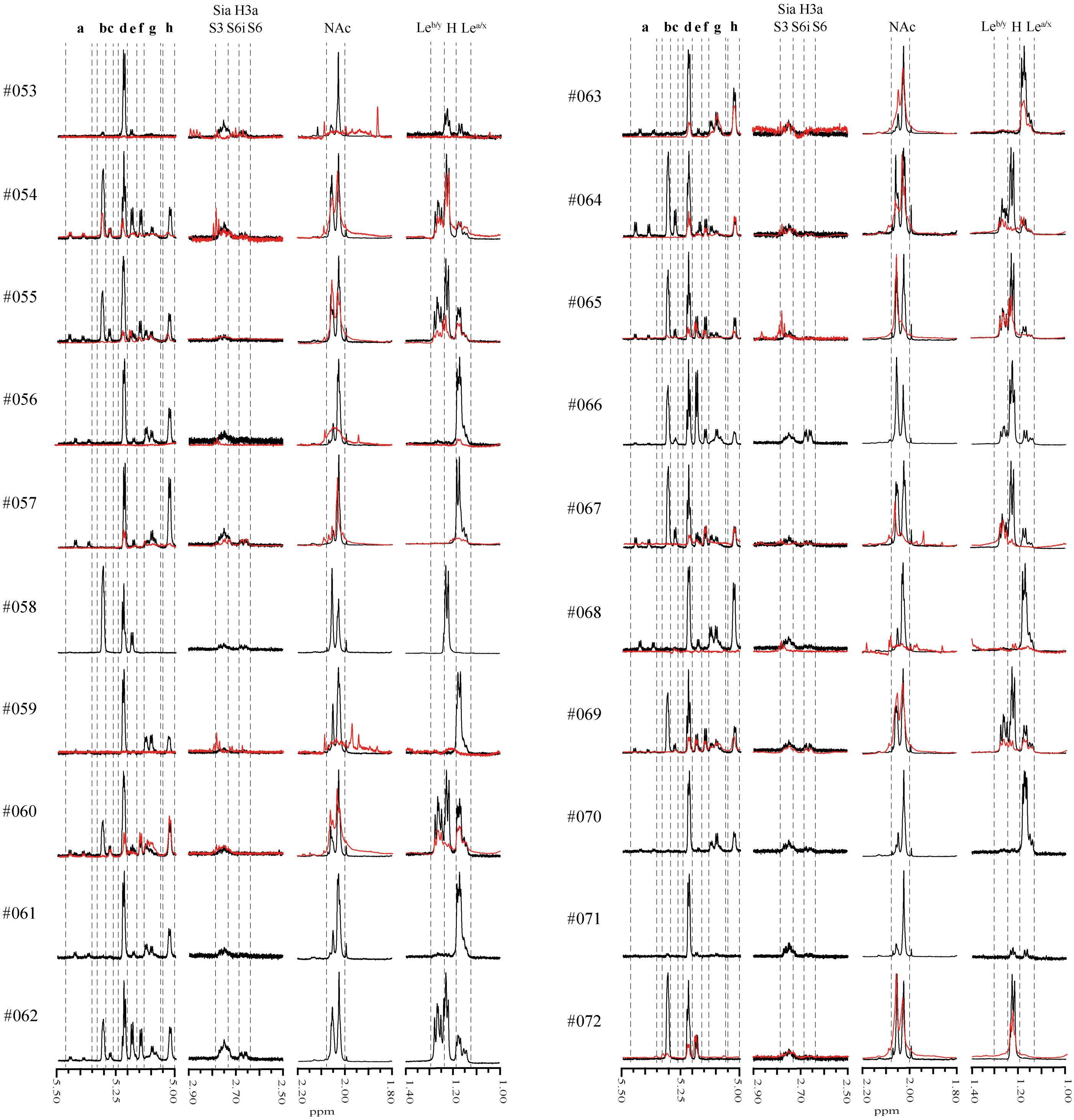
a bc d e f g h
Sia H3a
S3 S6i S6
NAc
Le$^{b/y}$ H Le$^{a/x}$
#053
#054
#055
#056
#057
#058
#059
#060
#061
#062
#063
#064
#065
#066
#067
#068
#069
#070
#071
#072
5.50
5.25
5.00
2.90
2.70
2.50
2.20
2.00
1.80
1.40
1.20
1.00
ppm

(Fig. S2 – part 4)

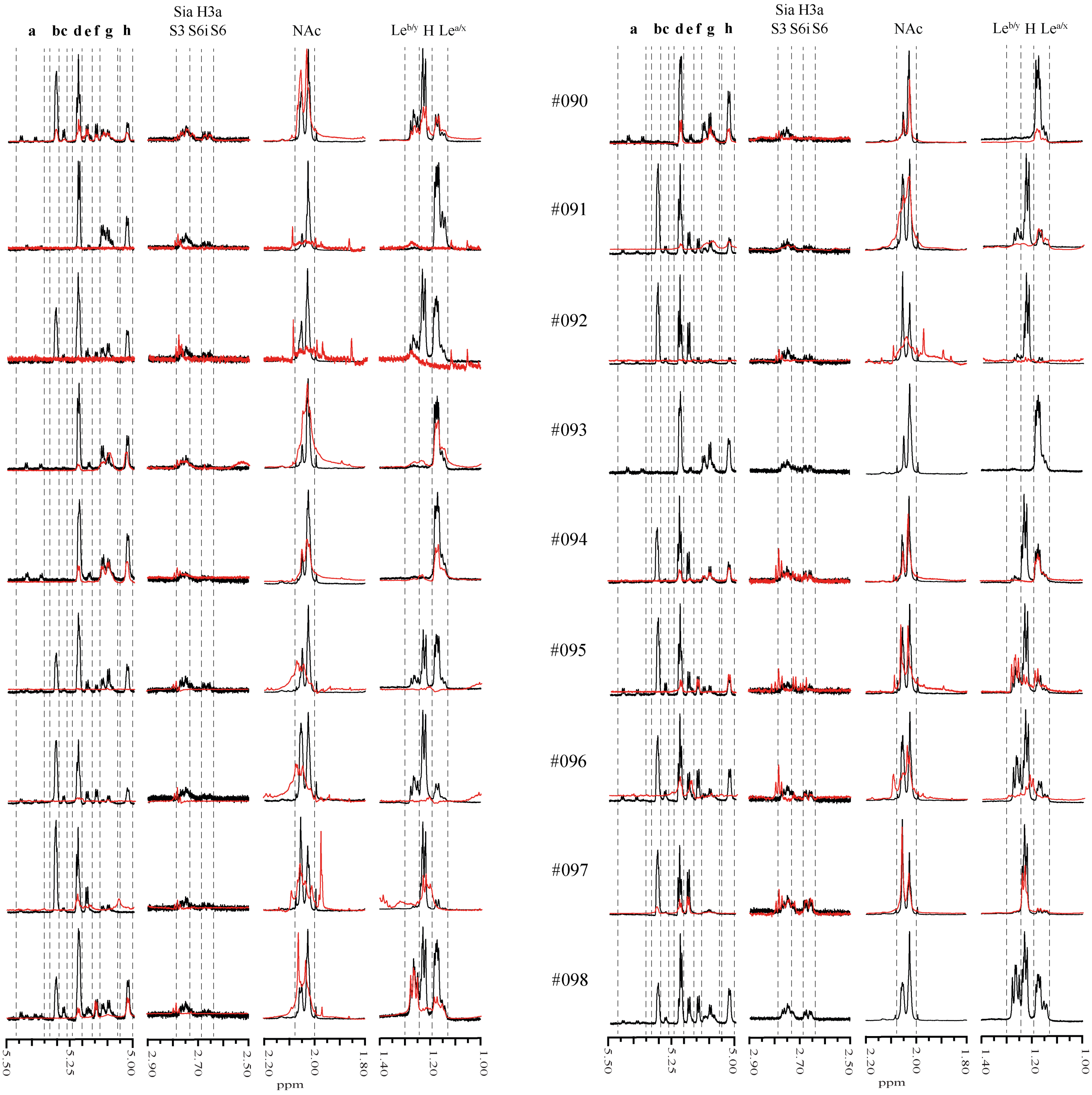
a bc d e f g h
Sia H3a
S3 S6i S6
NAc
Le$^{b/y}$ H Le$^{a/x}$
#081
#082
#083
#084
#085
#086
#087
#088
#089
#090
#091
#092
#093
#094
#095
#096
#097
#098
5.50 5.25 5.00
2.90 2.70 2.50
2.20 2.00 1.80
1.40 1.20 1.00
ppm

(Fig. S2 – part 5)

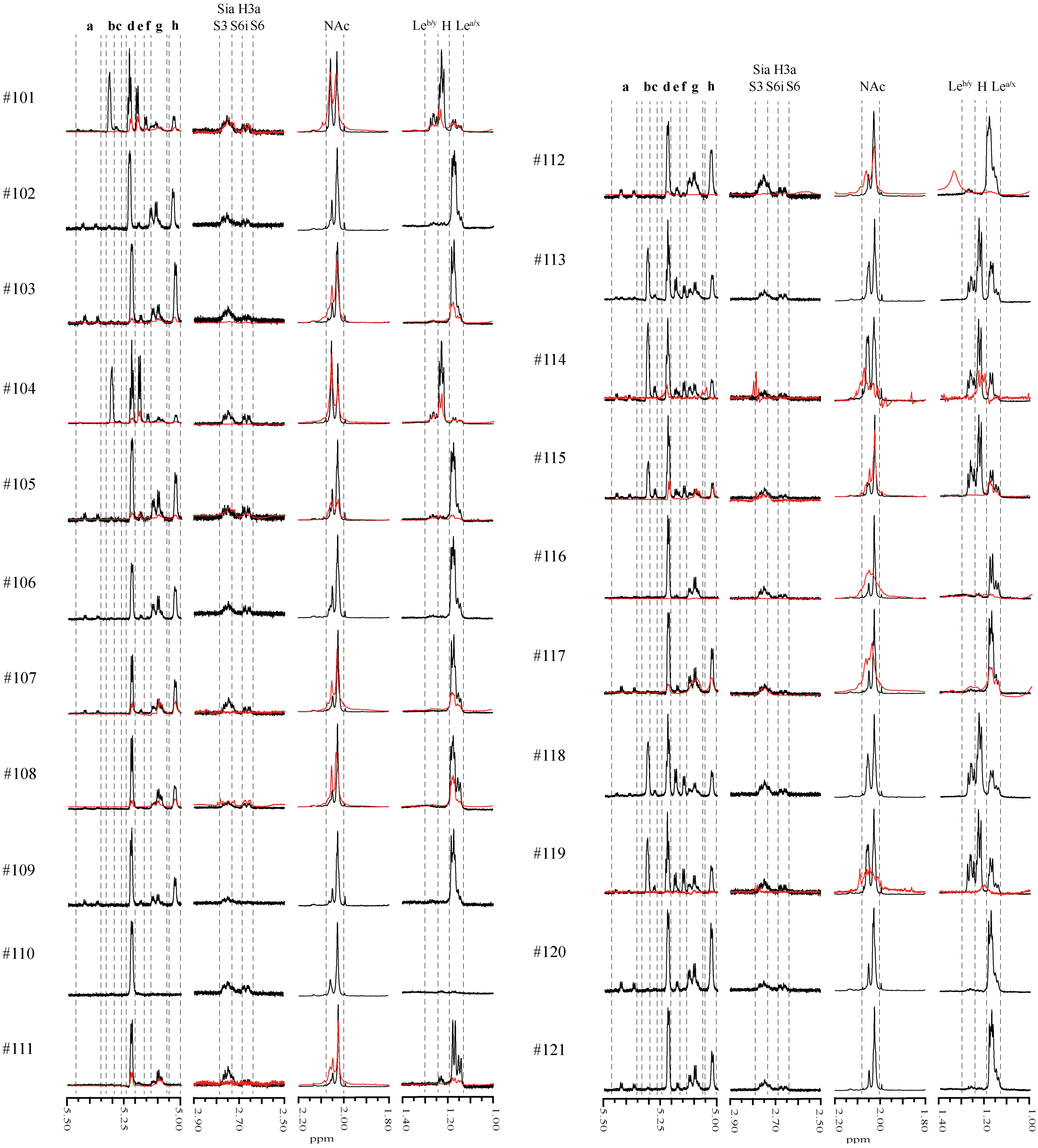
a bc d e f g h
Sia H3a
S3 S6i S6
NAc
Le b/y H Le a/x
#101
#102
#103
#104
#105
#106
#107
#108
#109
#110
#111
#112
#113
#114
#115
#116
#117
#118
#119
#120
#121
5.50 5.25 5.00
2.90 2.70 2.50
2.20 2.00 1.80
1.40 1.20 1.00
ppm

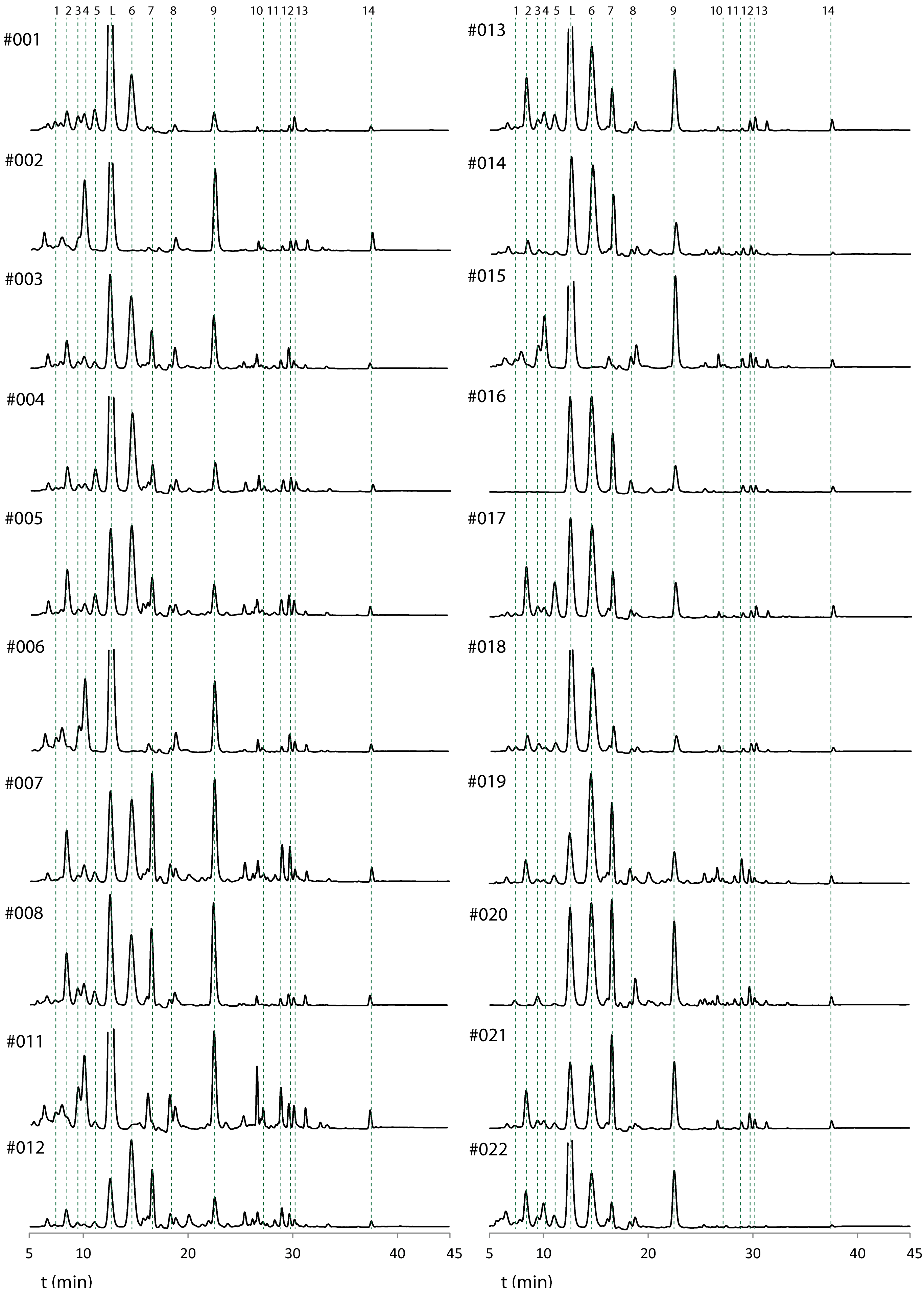

Fig. S3. HPAEC-PAD profiles of human milk oligosaccharides isolated from mothers' milk. Peaks for lactose (L) and reference structures 1-14 (Table 2, main paper) are indicated. Parts 1 – 5 show samples from 1. Ha Long Bay, 2. Phu Tho, 3. Tien Giang, 4. Ho Chi Mihn, 5. Ha Noi (Fig. S1).

(Fig. S3 – part2)

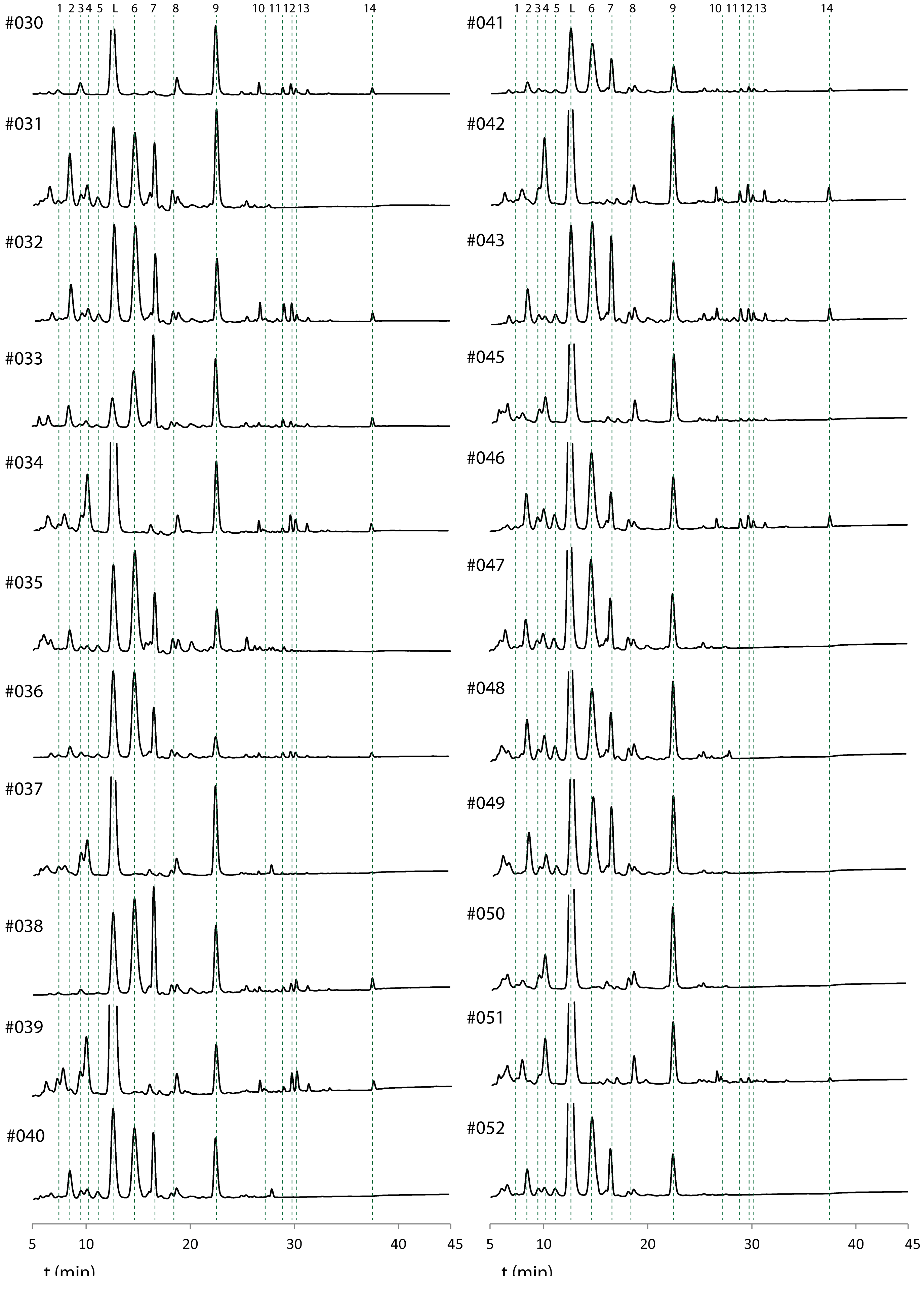
1 2 3 4 5 L 6 7 8 9 10 11 12 13 14
#030
#031
#032
#033
#034
#035
#036
#037
#038
#039
#040
#041
#042
#043
#045
#046
#047
#048
#049
#050
#051
#052
5 10 20 30 40 45
t (min)

(Fig. S3 – part 3)

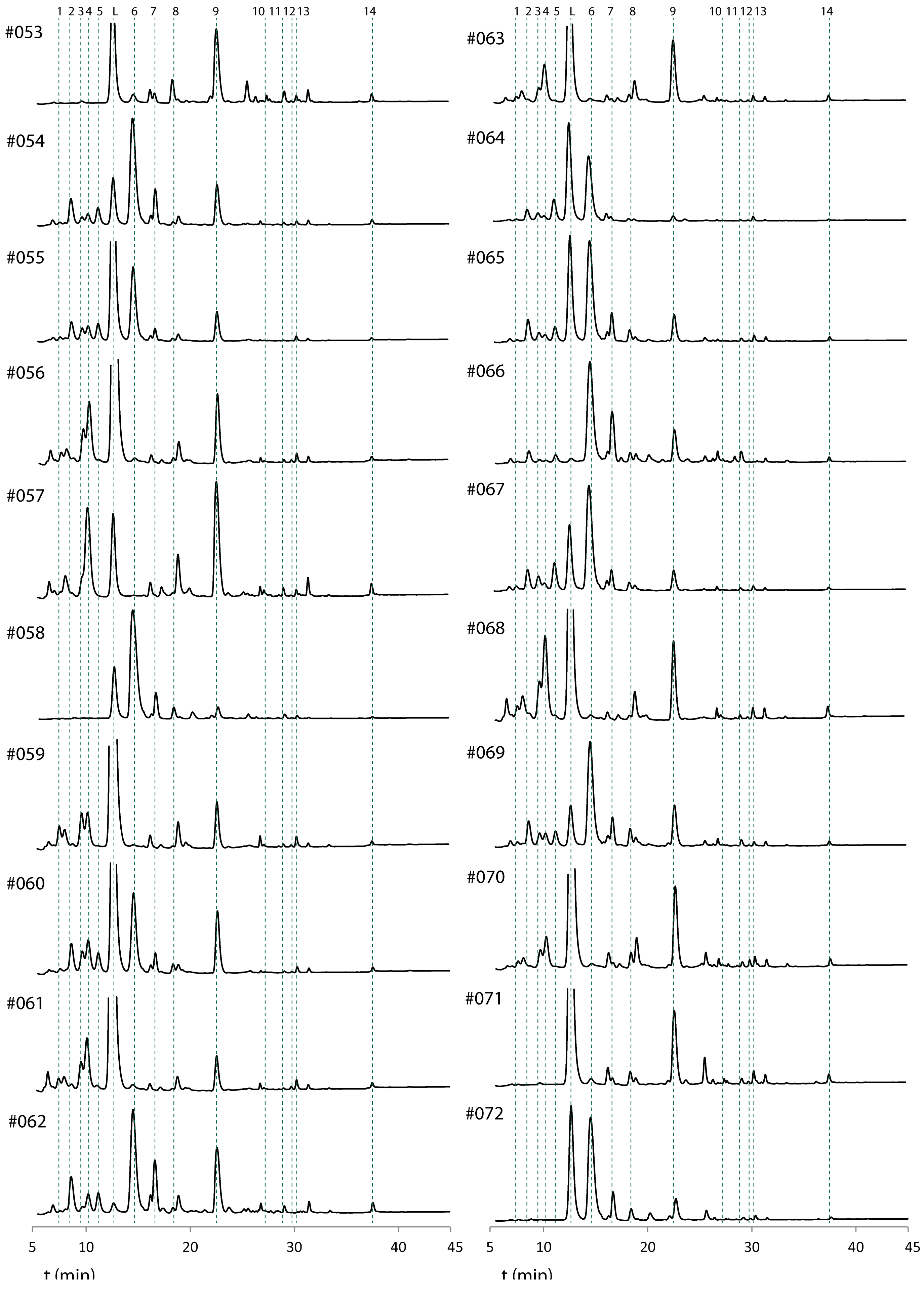
1 2 3 4 5 L 6 7 8 9 10 11 12 13 14
#053
#054
#055
#056
#057
#058
#059
#060
#061
#062
#063
#064
#065
#066
#067
#068
#069
#070
#071
#072
5
10
20
30
40
45
t (min)

(Fig. S3 – part 4)

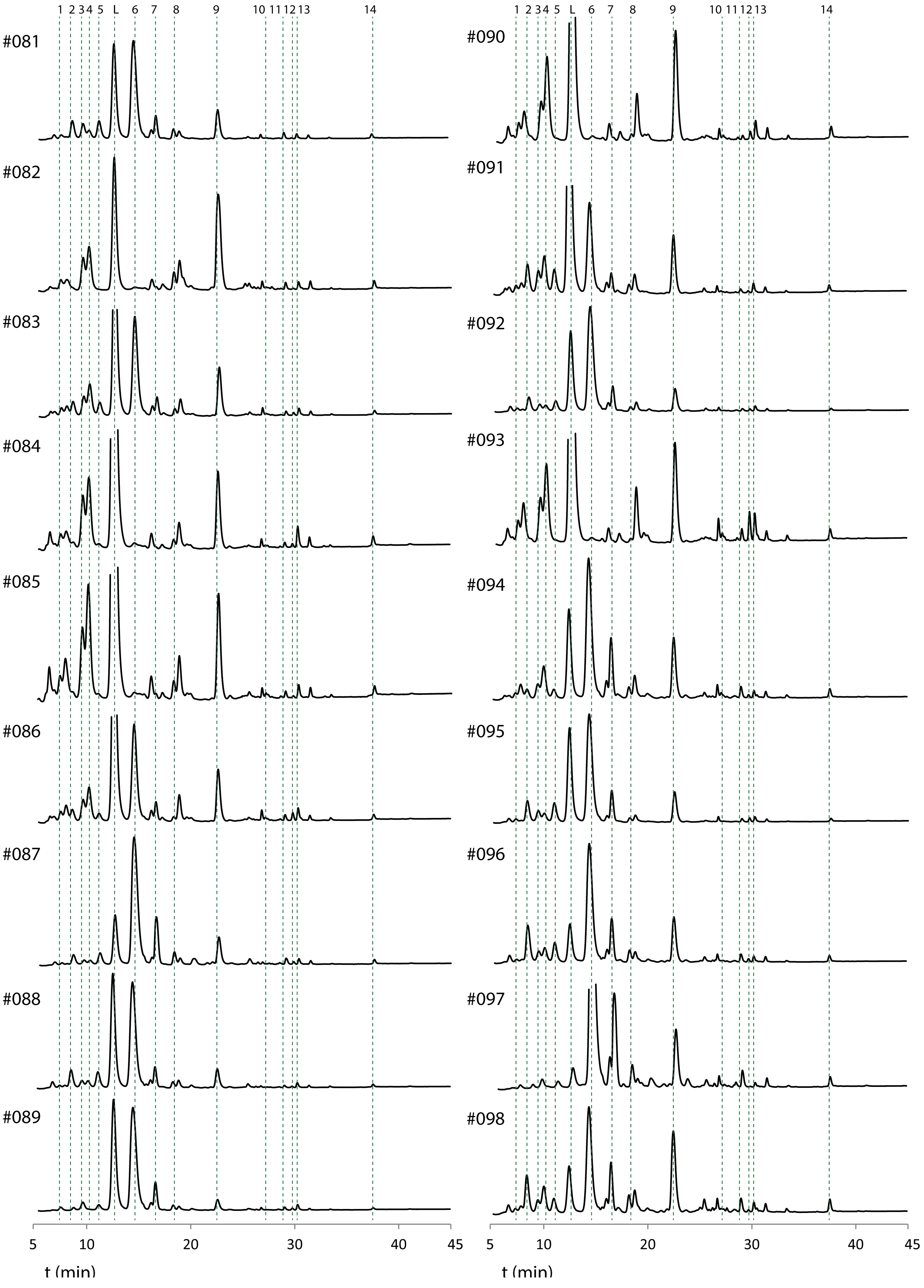
1 2 3 4 5 L 6 7 8 9 10 11 12 13 14
#081
#082
#083
#084
#085
#086
#087
#088
#089
#090
#091
#092
#093
#094
#095
#096
#097
#098
5 10 20 30 40 45
t (min)

(Fig. S3 – part 5)

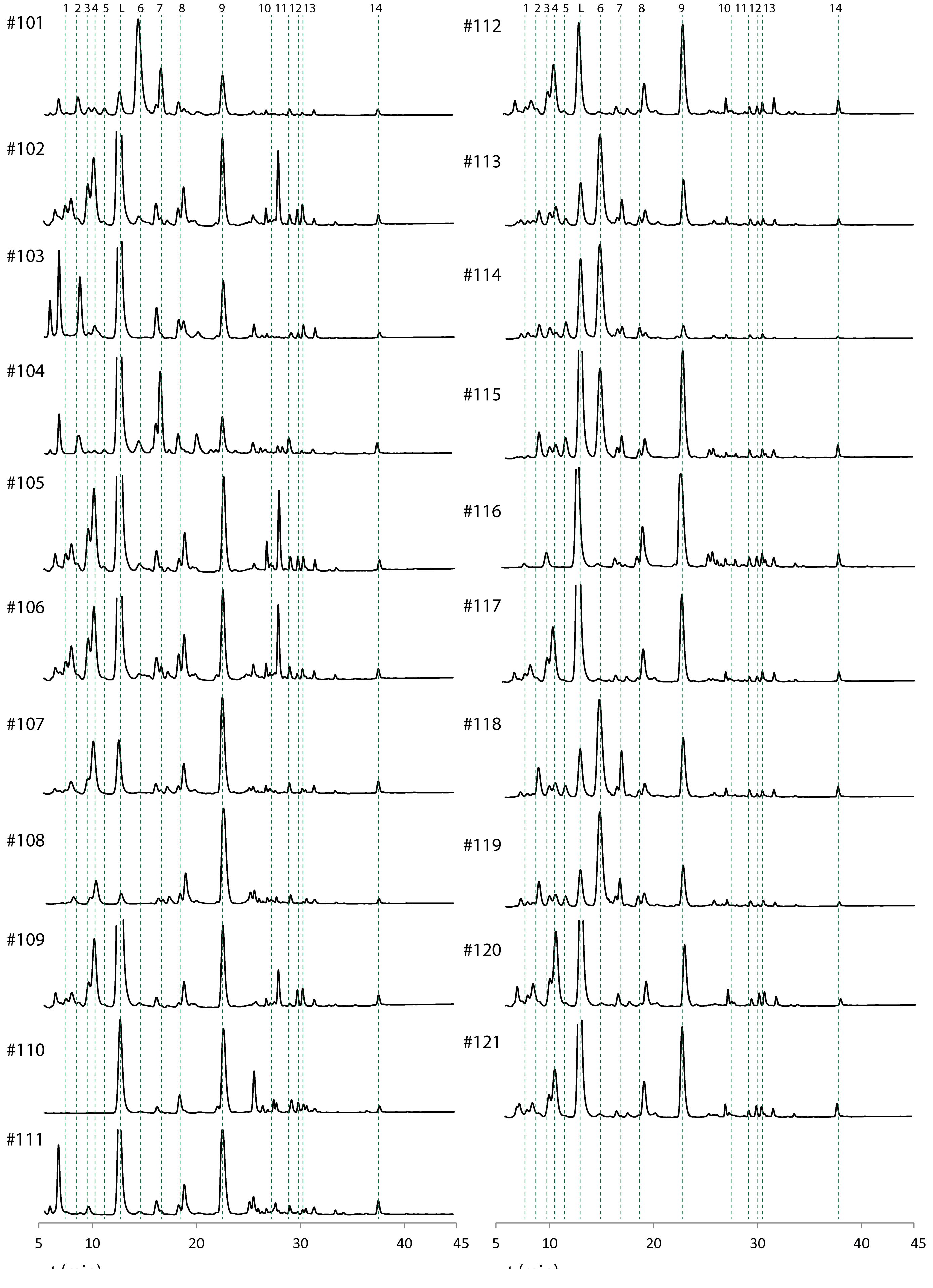
1 2 3 4 5 L 6 7 8 9 10 11 12 13 14
#101
#102
#103
#104
#105
#106
#107
#108
#109
#110
#111
#112
#113
#114
#115
#116
#117
#118
#119
#120
#121
5 10 20 30 40 45

Table S1. Levels of Lewis and Secretor epitopes obtained by 1D ^{1}H NMR analysis of hMOS from mothers' milk and corresponding infant faeces. Secretor/Lewis ratio (S/L) is based on Fuc CH_3 region integrals for isolated from milk samples and milk group (MG) classification based on NMR spectra and confirmed by HPAEC-PAD analysis.

	hMOS from milk						hMOS from faeces[a]								
M/F #	Le^a	Le^b	Le^x	Le^y	2′FL	H	Le^a	Le^b	Le^x	Le^y	2′FL	H	S/L[b]	MG	CP[c]
#001	0.12	0.10	0.32	0.10	0.34	0.02	0.04	0.60	0.01	0.00	0.00	1.00	1.06	1b	S
#002	0.57	0.00	0.43	0.00	0.00	0.00	N/A	N/A	N/A	N/A	N/A	N/A	0.00	2	Nd
#003	0.07	0.14	0.34	0.03	0.30	0.12	0.53	1.00	0.31	0.00	0.00	0.00	1.23	1b	S
#004	0.05	0.12	0.32	0.10	0.32	0.09	1.00	0.43	0.21	0.00	0.00	0.42	1.48	1b	S
#005	0.06	0.18	0.30	0.07	0.30	0.09	1.00	0.30	0.06	0.00	0.00	0.18	1.46	1b	S
#006	0.46	0.00	0.54	0.00	0.00	0.00	1.00	N/A	0.89	N/A	N/A	N/A	0.00	2	NS
#007	0.07	0.15	0.29	0.04	0.24	0.21	nd	nd	nd	nd	nd	nd	1.52	1a	Nd
#008	0.07	0.14	0.33	0.04	0.25	0.17	0.33	1.00	0.02	0.00	0.00	0.36	1.28	1b	S
#011	0.27	0.00	0.73	0.00	0.00	0.00	0.00	N/A	0.00	N/A	N/A	N/A	0.00	2	C
#012	0.03	0.11	0.26	0.04	0.33	0.23	1.00	0.67	0.29	0.08	0.00	0.65	1.89	1a	S
#013	0.07	0.15	0.29	0.06	0.33	0.10	0.00	0.00	0.00	0.00	0.00	0.00	1.55	1b	C
#014	0.01	0.08	0.21	0.02	0.44	0.24	0.00	0.00	0.00	0.00	0.00	0.00	2.44	1a	C
#015	0.41	0.00	0.59	0.00	0.00	0.00	1.00	N/A	0.62	N/A	N/A	N/A	0.00	2	C
#016	0.00	0.00	0.00	0.01	0.73	0.26	N/A	N/A	N/A	0.00	1.00	0.88	16.8	3b	NS
#017	0.03	0.14	0.27	0.11	0.35	0.10	nd	nd	nd	nd	nd	nd	1.74	1b	Nd
#018	0.02	0.08	0.24	0.05	0.51	0.10	0.93	0.83	0.00	0.00	0.00	1.00	2.11	1a	S
#019	0.02	0.10	0.20	0.04	0.39	0.25	1.00	0.93	0.27	0.09	0.00	0.05	2.50	1a	S
#020	0.00	0.00	0.24	0.00	0.41	0.35	N/A	N/A	0.00	N/A	0.00	1.00	2.54	3a	S
#021	0.03	0.13	0.28	0.03	0.27	0.26	1.00	0.23	0.15	0.00	0.00	0.09	1.93	1a	S
#022	0.14	0.14	0.32	0.05	0.28	0.07	0.00	0.00	0.00	0.00	0.00	0.00	1.08	1b	S
#030	0.00	0.00	0.97	0.00	0.00	0.03	nd	nd	nd	nd	nd	nd	0.00	4a	Nd
#031	0.10	0.16	0.33	0.03	0.24	0.14	1.00	0.35	0.44	0.11	0.00	0.19	1.20	1b	S
#032	0.06	0.12	0.28	0.02	0.35	0.17	0.78	0.43	0.46	0.21	0.06	1.00	1.73	1a	NS
#033	0.05	0.10	0.20	0.01	0.30	0.34	nd	nd	nd	nd	nd	nd	2.40	1a	Nd
#034	0.51	0.00	0.49	0.00	0.00	0.00	0.82	N/A	1.00	N/A	N/A	N/A	0.00	2	NS
#035	0.06	0.11	0.26	0.02	0.38	0.17	0.00	0.00	0.00	0.00	0.00	0.00	1.90	1a	C
#036	0.01	0.07	0.19	0.03	0.48	0.22	1.00	0.15	0.09	0.10	0.01	0.13	2.82	1a	S
#037	0.32	0.00	0.67	0.00	0.01	0.00	1.00	N/A	0.61	N/A	0.00	N/A	0.00	2	S
#038	0.00	0.00	0.14	0.00	0.48	0.38	N/A	N/A	1.00	N/A	0.00	0.45	6.90	3a	S
#039	0.39	0.00	0.61	0.00	0.00	0.00	1.00	N/A	1.00	N/A	N/A	N/A	0.00	2	NS
#040	0.04	0.12	0.27	0.03	0.32	0.22	1.00	0.41	0.02	0.53	0.00	0.21	1.62	1a	S
#041	0.01	0.10	0.26	0.03	0.38	0.22	1.00	0.08	0.14	0.00	0.00	0.04	2.05	1a	C
#042	0.52	0.00	0.48	0.00	0.00	0.00	nd	nd	nd	nd	nd	nd	0.00	2	Nd
#043	0.03	0.12	0.29	0.03	0.32	0.21	nd	nd	nd	nd	nd	nd	1.80	1a	Nd
#045	0.31	0.00	0.68	0.00	0.01	0.00	0.00	N/A	0.00	N/A	0.00	N/A	0.00	2	C
#046	0.09	0.14	0.28	0.05	0.33	0.11	0.00	0.00	0.00	0.00	0.00	0.00	1.30	1a	C
#047	0.07	0.12	0.25	0.04	0.37	0.15	nd	nd	nd	nd	nd	nd	1.57	1a	Nd
#048	0.09	0.14	0.34	0.05	0.25	0.13	nd	nd	nd	nd	nd	nd	1.07	1b	Nd
#049	0.10	0.13	0.28	0.02	0.32	0.15	1.00	0.71	0.99	0.77	0.36	1.18	1.57	1a	NS
#050	0.40	0.00	0.60	0.00	0.00	0.00	1.00	N/A	0.96	N/A	N/A	N/A	0.00	2	NS
#051	0.48	0.00	0.52	0.00	0.00	0.00	0.85	N/A	1.00	N/A	N/A	N/A	0.00	2	NS
#052	0.03	0.11	0.26	0.04	0.39	0.17	0.67	0.36	0.10	0.17	0.17	1.00	2.01	1a	S
#053	0.00	0.00	0.17	0.00	0.41	0.42	N/A	N/A	0.00	N/A	0.00	0.00	1.65	4a	C
#054	0.05	0.13	0.25	0.04	0.39	0.14	0.51	0.18	0.25	1.00	0.34	0.00	2.04	1a	NS
#055	0.11	0.13	0.25	0.07	0.38	0.06	0.40	0.21	0.28	0.10	0.02	1.00	1.38	1a	S
#056	0.49	0.00	0.51	0.00	0.00	0.00	0.00	N/A	0.00	N/A	N/A	N/A	0.01	2	C
#057	0.65	0.00	0.35	0.00	0.00	0.00	0.36	N/A	1.00	N/A	N/A	N/A	0.01	2	S
#058	0.00	0.00	0.00	0.00	0.84	0.16	nd	nd	nd	nd	nd	nd	34.3	3b	Nd
#059	0.31	0.00	0.69	0.00	0.00	0.00	0.00	N/A	0.00	N/A	N/A	N/A	0.00	2	C
#060	0.16	0.14	0.31	0.05	0.29	0.05	1.00	0.82	0.76	0.69	0.02	0.57	1.02	1b	S
#061	0.47	0.00	0.53	0.00	0.00	0.00	nd	nd	nd	nd	nd	nd	0.01	2	Nd
#062	0.07	0.16	0.30	0.03	0.25	0.19	nd	nd	nd	nd	nd	nd	1.50	1b	Nd
#063	0.53	0.00	0.47	0.00	0.00	0.00	1.00	N/A	0.96	N/A	N/A	N/A	0.01	2	S
#064	0.03	0.10	0.18	0.13	0.55	0.01	1.00	0.12	0.10	0.00	0.00	0.00	2.41	1a	S
#065	0.03	0.12	0.22	0.04	0.48	0.11	0.79	0.43	0.12	0.11	0.00	1.00	2.61	1a	S
#066	0.02	0.07	0.22	0.03	0.32	0.34	nd	nd	nd	nd	nd	nd	2.74	1a	Nd
#067	0.03	0.11	0.26	0.07	0.47	0.06	0.85	1.00	0.01	0.00	0.00	0.51	2.11	1a	S
#068	0.47	0.00	0.53	0.00	0.00	0.00	0.00	N/A	0.00	N/A	N/A	N/A	0.03	2	C
#069	0.05	0.14	0.30	0.03	0.38	0.10	1.00	0.28	0.27	0.23	0.00	0.50	1.57	1a	NS
#070	0.38	0.00	0.62	0.00	0.00	0.00	N/A	N/A	N/A	N/A	N/A	N/A	0.00	2	Nd
#071	0.00	0.00	0.38	0.00	0.38	0.24	nd	nd	nd	nd	nd	nd	1.43	4a	Nd
#072	0.00	0.00	0.00	0.00	0.81	0.19	N/A	N/A	N/A	N/A	0.06	1.00	192	3b	S

(Table S1 continued)

	hMOS from milk						hMOS from faeces								
	Le^a	Le^b	Le^x	Le^y	2´FL	H	Le^a	Le^b	Le^x	Le^y	2´FL	H	S/L	MG	CP
#081	0.04	0.10	0.29	0.04	0.45	0.08	1.00	0.33	0.39	0.25	0.14	0.92	1.81	1a	NS
#082	0.33	0.00	0.67	0.00	0.00	0.00	0.00	N/A	0.00	N/A	N/A	N/A	0.01	2	C
#083	0.15	0.08	0.31	0.04	0.34	0.08	0.00	0.00	0.00	0.00	0.00	0.00	0.95	1a	C
#084	0.37	0.00	0.63	0.00	0.00	0.00	1.00	N/A	0.88	N/A	N/A	N/A	0.02	2	NS
#085	0.42	0.00	0.58	0.00	0.00	0.00	0.71	N/A	1.00	N/A	N/A	N/A	0.01	2	NS
#086	0.19	0.05	0.34	0.03	0.29	0.10	0.00	0.00	0.50	1.00	0.00	0.00	0.72	1a	C
#087	0.02	0.11	0.20	0.07	0.49	0.11	1.00	0.00	0.00	0.29	0.00	0.00	2.57	1a	C
#088	0.00	0.00	0.10	0.04	0.68	0.18	N/A	N/A	0.14	0.00	0.00	1.00	4.65	3a	S
#089	0.17	0.11	0.33	0.05	0.27	0.07	0.67	1.00	0.23	0.00	0.00	0.43	0.81	1b	S
#090	0.45	0.00	0.55	0.00	0.00	0.00	0.96	N/A	1.00	N/A	N/A	N/A	0.01	2	NS
#091	0.04	0.07	0.21	0.02	0.56	0.10	1.00	0.00	0.30	0.00	0.00	0.00	2.78	1a	S
#092	0.03	0.02	0.09	0.01	0.63	0.22	0.00	0.00	0.00	0.00	0.00	0.00	6.32	1a	C
#093	0.36	0.00	0.64	0.00	0.00	0.00	nd	nd	nd	nd	nd	nd	0.00	2	Nd
#094	0.16	0.02	0.22	0.01	0.42	0.17	1.00	0.00	0.73	0.00	0.00	0.12	1.49	1a	S
#095	0.04	0.09	0.22	0.05	0.49	0.11	1.00	0.59	0.00	0.00	0.00	0.00	2.38	1a	S
#096	0.05	0.15	0.27	0.03	0.37	0.13	0.00	0.00	0.15	0.12	0.00	1.00	1.87	1a	S
#097	0.00	0.00	0.10	0.01	0.57	0.32	N/A	N/A	0.30	0.51	0.28	1.00	6.57	3a	NS
#098	0.11	0.14	0.36	0.02	0.24	0.13	nd	nd	nd	nd	nd	nd	1.10	1b	Nd
#101	0.04	0.08	0.20	0.02	0.41	0.25	1.00	0.38	0.47	0.50	0.02	0.60	2.75	1a	S
#102	0.39	0.00	0.59	0.00	0.02	0.00	nd	nd	nd	nd	nd	nd	0.07	2	Nd
#103	0.57	0.00	0.43	0.00	0.00	0.00	1.00	N/A	1.33	N/A	N/A	N/A	0.03	2	S
#104	0.01	0.05	0.13	0.02	0.40	0.39	0.00	1.00	0.50	0.00	0.04	0.95	5.00	1a	S
#105	0.39	0.00	0.61	0.00	0.00	0.00	1.00	N/A	0.72	N/A	N/A	N/A	0.09	2	NS
#106	0.41	0.00	0.58	0.00	0.01	0.00	nd	nd	nd	nd	nd	nd	0.03	2	Nd
#107	0.52	0.00	0.48	0.00	0.00	0.00	0.96	N/A	1.00	N/A	N/A	N/A	0.02	2	NS
#108	0.39	0.00	0.61	0.00	0.00	0.00	1.00	N/A	0.96	N/A	N/A	N/A	0.02	2	NS
#109	0.50	0.00	0.50	0.00	0.00	0.00	nd	nd	nd	nd	nd	nd	0.02	2	Nd
#110	0.00	0.00	0.00	0.00	0.00	0.00	nd	nd	nd	nd	nd	nd	1.00	4b	Nd
#111	0.00	0.00	0.99	0.00	0.01	0.00	N/A	N/A	1.00	N/A	0.00	N/A	0.08	4a	NS
#112	0.38	0.00	0.62	0.00	0.00	0.00	0.00	N/A	0.00	N/A	N/A	N/A	0.05	2	C
#113	0.10	0.08	0.32	0.02	0.36	0.12	nd	nd	nd	nd	nd	nd	1.21	1a	Nd
#114	0.03	0.10	0.29	0.06	0.47	0.05	0.00	0.00	0.00	0.00	0.00	1.00	1.75	1a	S
#115	0.08	0.10	0.29	0.07	0.41	0.05	1.00	0.00	0.23	0.00	0.00	0.00	1.55	1a	S
#116	0.00	0.00	0.99	0.00	0.00	0.01	N/A	N/A	0.00	N/A	N/A	0.00	0.00	4a	C
#117	0.50	0.00	0.50	0.00	0.00	0.00	0.79	N/A	1.00	N/A	N/A	N/A	0.02	2	NS
#118	0.07	0.11	0.28	0.02	0.37	0.15	nd	nd	nd	nd	nd	nd	1.69	1a	Nd
#119	0.06	0.13	0.33	0.03	0.35	0.10	0.00	0.00	0.00	0.00	0.00	0.00	1.38	1a	C
#120	0.48	0.00	0.51	0.00	0.01	0.00	nd	nd	nd	nd	nd	nd	0.01	2	Nd
#121	0.45	0.00	0.54	0.00	0.01	0.00	nd	nd	nd	nd	nd	nd	0.03	2	Nd

[a.] Faecal data are expressed in ratio relative to the highest remaining epitope compared to milk epitopes; 1.00 indicated the highest intensity peak., nd indicates that no faecal sample was available, N/A indicated the structural epitope was already not present in the milk.

[b.] Se (Secretory activity) levels are derived from the integral δ 1.19-1.24 adding ½ of the integral δ 1.24-1.30; the Le (Lewis activity) levels are similarly derived from the integral δ 1.14-1.19 adding ½ of the integral δ 1.24-1.30.

[c.] Consumption patterns are derived from the difference between hMOS found in the milk and in the faeces. C indicated complete consumption of all hMOS, S signifies consumption of one or more specific epitopes, while other epitopes remain in the faeces. NS indicates samples where all structural elements found in the milk hMOS are still observed in the hMOS isolated from the faeces. For sample sets lacking a faecal sample nd is used.

Table S2. Food intake questionnaire at week 1 and week 4 of age. BM – breastmilk, FF – Formula, LQ – other liquids, water, juice, milk etc. and SF – solid foods. "Y" = yes, "N" = no and "-" = no data

	Week 1		Week 4			
M/F#	BM	FF	BM	FF	LQ	SF
#001	Y	N	Y	N	N	Y
#002	Y	N	Y	N	N	N
#003	Y	N	Y	Y	Y	N
#004	-	-	-	-	-	-
#005	Y	N	Y	N	N	N
#006	Y	N	Y	N	N	Y
#007	Y	Y	Y	Y	N	N
#008	Y	N	Y	N	N	N
#011	Y	N	Y	Y	N	N
#012	Y	N	Y	Y	N	N
#013	Y	N	Y	N	N	N
#014	Y	N	Y	Y	N	N
#015	Y	N	Y	N	N	N
#016	-	-	-	-	-	-
#017	Y	N	Y	N	N	N
#018	Y	Y	Y	N	N	N
#019	Y	N	Y	N	N	N
#020	Y	N	Y	N	N	N
#021	Y	N	Y	N	N	N
#022	Y	N	Y	N	N	N
#030	Y	N	Y	N	N	N
#031	Y	N	Y	Y	N	N
#032	Y	N	Y	Y	N	N
#033	Y	N	Y	N	N	N
#034	Y	N	Y	N	N	N
#035	Y	N	Y	N	N	N
#036	Y	N	Y	N	N	N
#037	Y	N	Y	N	Y	N
#038	Y	N	Y	N	Y	N
#039	Y	N	Y	N	N	N
#040	Y	N	Y	N	N	N
#041	Y	N	Y	Y	N	N
#042	Y	N	Y	N	N	N
#043	Y	N	Y	N	N	N
#045	-	-	-	-	-	-
#046	Y	N	Y	N	N	N
#047	Y	N	Y	N	N	N
#048	Y	N	Y	N	N	N
#049	Y	N	Y	N	N	N
#050	-	-	-	-	-	-
#051	Y	N	Y	N	N	N
#052	Y	N	Y	N	N	N
#053	Y	N	Y	N	N	N
#054	Y	N	Y	N	N	N
#055	Y	N	Y	N	N	N
#056	Y	N	Y	N	N	N
#057	Y	N	Y	N	N	N
#058	Y	N	Y	N	N	N
#059	Y	N	Y	N	N	N
#060	Y	N	Y	N	N	N
#061	Y	N	Y	N	N	N
#062	Y	N	Y	N	N	N
#063	Y	N	Y	N	N	N
#064	Y	N	Y	N	N	N
#065	Y	N	Y	N	N	N
#066	Y	N	Y	N	N	N
#067	Y	N	Y	Y	N	N
#068	Y	N	Y	N	N	N
#069	Y	N	Y	N	N	N
#070	Y	N	Y	N	N	N
#071	Y	N	Y	N	N	N
#072	Y	N	Y	N	N	N

(Table S2. Continued)

	Week 1		Week 4			
M/F#	BM	FF	BM	FF	LQ	SF
#081	Y	N	Y	Y	N	N
#082	Y	N	Y	N	N	N
#083	Y	N	Y	Y	N	N
#084	Y	Y	Y	N	N	N
#085	Y	N	Y	N	N	N
#086	Y	Y	Y	Y	N	N
#087	Y	N	Y	N	N	N
#088	Y	N	Y	Y	N	N
#089	Y	N	Y	N	N	N
#090	Y	N	Y	N	N	N
#091	Y	N	Y	N	N	N
#092	-	-	-	-	-	-
#093	Y	N	Y	N	N	N
#094	Y	N	Y	N	N	N
#095	Y	N	Y	N	N	N
#096	Y	N	Y	N	N	N
#097	Y	N	Y	N	N	N
#098	Y	N	Y	Y	N	N
#101	Y	N	Y	N	N	N
#102	Y	N	Y	N	N	N
#103	Y	N	Y	N	N	N
#104	Y	N	Y	N	N	N
#105	Y	N	Y	N	N	N
#106	-	-	-	-	-	-
#107	Y	N	Y	N	N	N
#108	Y	N	Y	Y	N	N
#109	Y	N	Y	N	N	N
#110	-	-	-	-	-	-
#111	Y	Y	Y	Y	N	N
#112	Y	Y	Y	Y	N	N
#113	Y	N	Y	N	N	N
#114	Y	N	Y	N	N	N
#115	Y	N	Y	N	N	N
#116	Y	N	Y	N	N	N
#117	-	-	-	-	-	-
#118	Y	N	Y	N	N	N
#119	Y	N	Y	N	N	N
#120	Y	N	Y	N	N	N
#121	Y	N	Y	N	N	N
